# Metabolite-induced DNA damage drives stochastic stem cell loss and clonal hematopoiesis

**DOI:** 10.1016/j.stem.2026.02.011

**Published:** 2026-04-02

**Authors:** Ashley N. Kamimae-Lanning, Jill M. Brown, Matthias Günther, Franziska Esau, Holly Russell, Lise Larcher, Frédéric Langevin, Tomoya Isobe, Nicola K. Wilson, Felix A. Dingler, Rebecca L. Cordell, Meng Wang, Christopher L. Millington, Nina Claudino, Ewa Gogola, Matthew Nicholls, Verena Körber, Berthold Göttgens, Marella F.T.R. de Bruijn, Juan I. Garaycoechea, Jean Soulier, Thomas Höfer, Ketan J. Patel

**Affiliations:** 1 University of Oxford, MRC Weatherall Institute of Molecular Medicine, Molecular Haematology Unit, Oxford, UK; 2 Division of Theoretical Systems Biology, German Cancer Research Center, Heidelberg, Germany; 3 Institut de Recherche Saint-Louis (IRSL), Université Paris Cité, 75010 Paris, France; 4 INSERM UMR1342 CNRS EMR8000, Paris, France; 5 Saint Louis Hospital, Hematology Laboratory, APHP, Paris, France; 6 Centre de Référence Maladies Rares “Aplasie Médullaire”, Saint-Louis and Robert Debré Hospitals, Paris, France; 7 MRC Laboratory of Molecular Biology, Cambridge, UK; 8 Cambridge Stem Cell Institute, Department of Haematology, University of Cambridge, Cambridge, UK; 9 Centre for Environmental Health and Sustainability, The University of Leicester, Leicester, UK; 10 Division of Nutritional Sciences, Cornell University, Ithaca, NY, USA; 11 Department of Haematology, Addenbrooke's Hospital, Cambridge, UK; 12 Hubrecht Institute, Utrecht, the Netherlands

**Keywords:** clonal hematopoiesis, HSC attrition, bone marrow failure, endogenous DNA damage, formaldehyde, Fanconi anemia, neutral drift, stem cell aging, somatic evolution

## Abstract

DNA damage and mutations in hematopoietic stem cells (HSCs) enable clonal hematopoiesis (CH). Such damage occurs across a lifetime, but its origins remain unknown. Here, we demonstrate that endogenous formaldehyde causes HSC attrition and subsequently CH. We generated conditional mouse models lacking formaldehyde detoxification and Fanconi anemia (FA) DNA repair in blood. Formaldehyde protection was crucial for embryonic HSC emergence and throughout life. Despite severe deficiencies in HSCs, these mice produced blood for many months. To determine what enables this, we employed an unbiased method for detecting clones, which exploits somatic variant data. This revealed initial polyclonal hematopoiesis that diminishes to monoclonal hematopoiesis, devoid of known genetic selection. Furthermore, in FA children, we find the same transition to monoclonal hematopoiesis. Therefore, DNA damage-induced attrition down to the last functional cell can be a driving force for CH, representing an alternative route to CH other than purely by fitness-enhancing selection.

## Highlights

• Endogenous formaldehyde diminishes HSPC numbers from embryonic development onward
• Severely depleted HSPC pools can sustain blood production until stochastic collapse
• Stem cell loss alone can give rise to clonal hematopoiesis, without driver mutations
• Similar attrition-driven clonal patterns appear in the Fanconi anemia setting

## Graphical abstract

**Figure:**
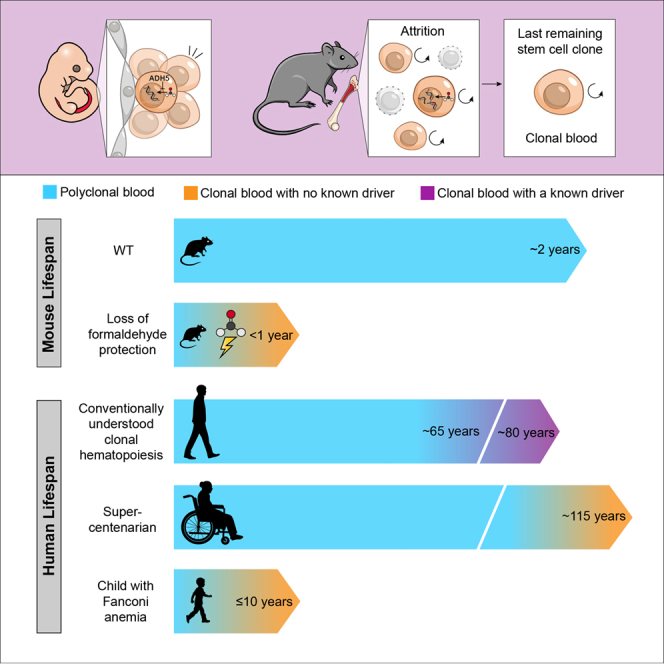

## Teaser

Protection against endogenous formaldehyde is important for blood stem cells throughout life. By studying blood-specific knockout mice and Fanconi anemia patients, Kamimae-Lanning and colleagues reveal that clonal hematopoiesis can arise not just through selective drivers but also through stochastic stem cell attrition, uncovering an alternative pathway shaping blood aging and disease.

## Introduction

Acquisition of somatic mutations with age results in tissue mosaicism and can lead to the expansion of competitively advantageous cells.^1^^,^^2^^,^^3^^,^^4^^,^^5^ In blood, this often leads to clonal hematopoiesis (CH), impairing blood production, and increasing disease risk.^6^^,^^7^ DNA damage and error-prone repair are key drivers of somatic mosaicism. The DNA damage response (DDR) contributes to stem cell aging, while mis-repair creates mutations that can give altered stem cells a competitive advantage. Chemotherapy accelerates CH by selecting stem cells carrying mutations in DDR such as *TP53*, *PPM1D*, and *CHEK2*.^8^^,^^9^ Similarly, inherited conditions affecting DNA repair, such as in Fanconi anemia (FA), hasten bone marrow failure (BMF) and clonal malignancies by accelerating genomic instability.^10^ While much research focuses on genetic changes driving clonal dominance, a large percentage of individuals with CH do not harbor any known driver mutations.^11^^,^^12^^,^^13^ This suggests that there must be other yet to be discovered mechanisms by which CH can arise.

Key insights come from whole-genome sequencing (WGS) of blood and bone marrow (BM)-derived colonies, which reveal that mutational signatures increase with age but can begin *in utero*, with some driver mutations for adult myeloproliferative diseases emerging prenatally.^12^^,^^14^^,^^15^^,^^16^^,^^17^ Comparisons between hematopoietic stem cells (HSCs) and their progeny show minimal differences in their mutational loads, indicating that processes independent of DNA replication can cause mutagenesis.^18^^,^^19^^,^^20^^,^^21^

An important question is what drives DNA damage and mutations in somatic cells, from HSCs to non-dividing neurons.^15^^,^^18^^,^^19^^,^^22^ Decay of DNA caused by oxygen and water is perhaps the main source of this damage,^23^ but reactive metabolites also adduct DNA. Aldehydes such as acetaldehyde (from ethanol metabolism) and formaldehyde (a byproduct of various metabolic processes such as demethylation) are an important class of endogenous metabolites that attack DNA.^24^^,^^25^^,^^26^ Mammals possess a two-tier protection mechanism preventing formaldehyde from causing significant DNA damage. Tier 1 comprises two enzymes that clear formaldehyde (principally ADH5 but also ALDH2). The second tier of protection consists of two DNA repair pathways that reverse formaldehyde-induced DNA base and protein crosslinks; loss of these repair mechanisms in humans causes FA, where HSC depletion and cancer risk predominate, or Cockayne syndrome, which causes striking premature aging.^25^^,^^27^^,^^28^

Here, we set out to define when and where protection against endogenous formaldehyde genotoxicity operates and how this shapes hematopoiesis. We establish that this protection matters from HSC emergence in embryogenesis through to post-natal life. Furthermore, we show that formaldehyde-induced HSPC attrition culminates in monoclonal hematopoiesis, defining stochastic attrition as a mechanism for the emergence of CH.

## **Results**

### Formaldehyde protection is important in HSCs, starting from their emergence in the embryo

**Figure 1:**
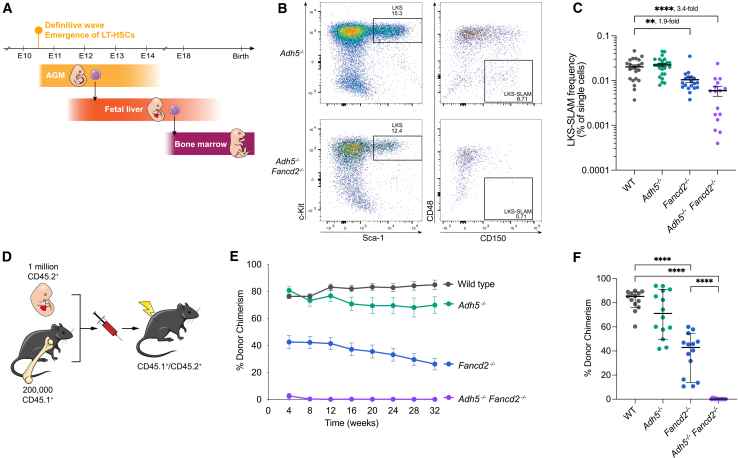
HSCs lacking formaldehyde protection are depleted at E13.5 (A) Definitive HSC locations during murine embryogenesis (E, days post-conception; LT-HSCs, long-term HSCs; AGM, aorta-gonad-mesonephros). (B) Flow cytometry gating of LKS-SLAM (Lin^−^ c-Kit^+^ Sca-1^+^ CD41^−^ CD48^−^ CD150^+^) cells (immunophenotypic HSCs) in E13.5 fetal livers. (C) Flow cytometry quantification of LKS-SLAM HSCs in E13.5 fetal livers (1 dot per liver, *n* = 22, 25, 19, 16, left to right, mean ± SEM). (D) Schematic depicting competitive transplantation assay. (E) Donor chimerism from monthly peripheral blood across 32 weeks post-transplant (recipient numbers: *n*_*WT*_ = 13, *n*_*Adh5*_ = 14, *n*_*Fancd2*_ = 14, *n*_*Adh5 Fancd2*_ = 14, mean ± SEM). (F) Donor chimerism at week 16 (earliest time point to assay for long-term reconstitutive capability) (*n*_*recipients*_ as in E, mean ± SEM shown). *p* values were determined by two-tailed Mann-Whitney U test, ^∗^*p* < 0.05, ^∗∗^*p* < 0.01, ^∗∗∗^*p* < 0.001 ^∗∗∗∗^*p* < 0.0001. See also Figure S1.

We have previously shown that *Adh5*^−/−^ *Fancd2*^−/−^ mice constitutively lacking ADH5 (the main formaldehyde-detoxifying enzyme) and FANCD2 (a key FA DNA crosslink repair protein) die within 2 months of birth due to a collapse in blood production and widespread DNA damage. These animals have a profound deficiency of HSCs.^25^ That these animals manifest profound HSC attrition within 3–7 weeks of age prompted us to ask when they begin to diminish. Definitive HSCs begin their segregation within the dorsal aortic endothelium at embryonic day (E)9.5 and emerge as pre-HSCs from the dorsal aorta around E10.5; these early HSCs then migrate to the fetal liver where further maturation and significant expansion occurs between E12 and E16.^29^^,^^30^^,^^31^^,^^32^^,^^33^^,^^34^ A final migration step at E17.5 via the blood begins to seed the BM, the mature site of blood production (Figure 1A).^35^ We assessed the number of immunophenotypic HSCs (Lin^−^ c-Kit^+^ Sca-1^+^ CD41^−^ CD48^−^ CD150^+^; LKS-SLAM) present in the fetal liver at E13.5 in *Adh5*^−/−^ *Fancd2*^−/−^ C57BL/6 × 129S6/Sv hybrid embryos (Figures 1B and 1C), which are obtained at sub-Mendelian ratios (Figures S1A and S1B). This shows a significant depletion of HSCs, which are 3.4-fold lower than that seen in wild type (WT) (Figure 1C). The functional activity of these HSCs, as assessed by conducting competitive long-term repopulation of *Adh5*^−/−^ *Fancd2*^−/−^ fetal liver cells in irradiated CD45.1/2 recipients, showed a profound inability to reconstitute hematopoiesis (Figures 1E and 1F). Together, these data indicate that formaldehyde protection is important for hematopoiesis during development, from the point when HSCs expand in the fetal liver.

**Figure 2:**
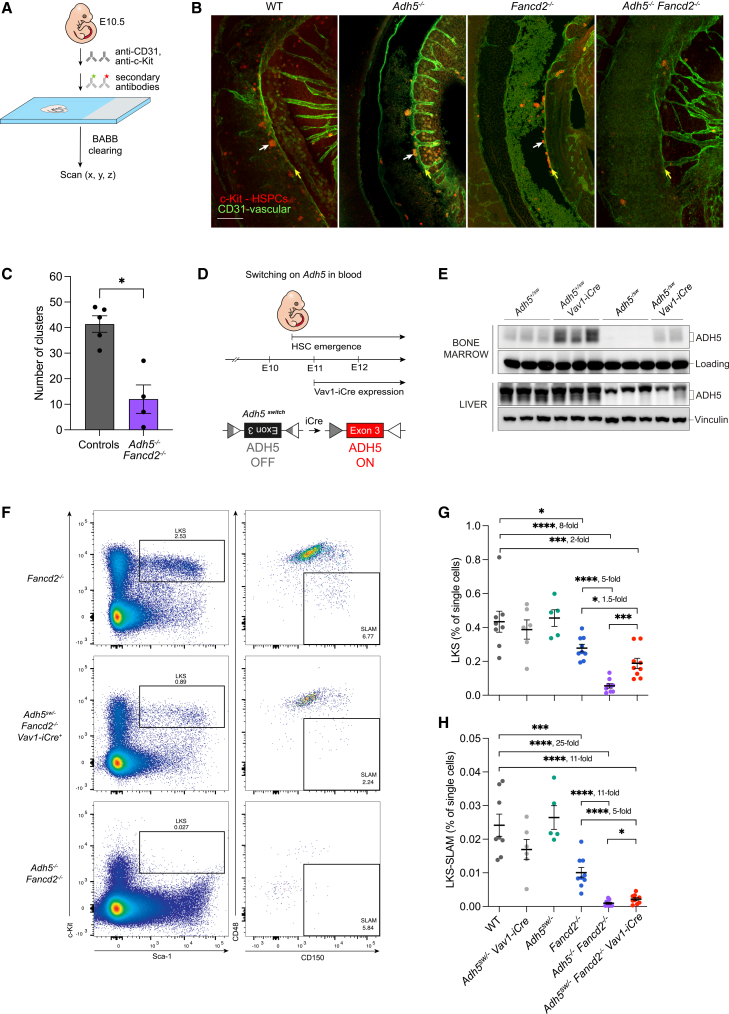
Formaldehyde protection matters at the point of HSC emergence (A) Experimental outline to observe hematopoietic clusters emerging from the dorsal aorta. (B) Whole-mount microscopy orthogonal composite projections of E10.5 dorsal aortae in *Adh5*^−/−^*Fancd2*^−/−^ and controls, showing c-Kit^+^ hematopoietic cells (red) and CD31^+^ vasculature (green). The white arrow indicates an example hematopoietic cluster emerging from the ventral aspect of the dorsal aorta (yellow arrows). Scale bar, 100 μm. (C) Numbers of emerging hematopoietic clusters in contact with the ventral aspect of the dorsal aorta from *Adh5*^−/−^*Fancd2*^−/−^ and genetic control E10.5 embryos (*n*_*control*_ = 5, *n*_*Adh5 Fancd2*_ = 4, mean ± SEM). (D) *Adh5*^*switch*^*(Adh5*^*sw*^*)* conditional mouse: from E11, the *Vav1* promoter drives expression of iCre recombinase in blood cells; iCre recombination between self-inactivating *loxP* sites flanking an inverted critical exon of *Adh5*, restoring a functional *Adh5*. (E) Western blot of ADH5 in BM and liver from *Adh5*^*sw/+*^, *Adh5*^*sw/+*^*Vav1-iCre*, *Adh5*^*sw/−*^, and *Adh5*^*sw/−*^*Vav1-iCre* mice with non-specific antibody binding (BM) or vinculin (liver) as loading controls. (F) Single representative flow cytometry plots of BM LKS (Lin^*−*^ c-Kit^+^ Sca-1^+^) and LKS-SLAM (immunophenotypic HSCs) analysis from *Fancd2*^−/−^, *Adh5*^*sw/−*^*Fancd2*^−/−^*Vav1-iCre*, and *Adh5*^−/−^*Fancd2*^−/−^*Vav1-iCre* mice. (G) Quantification of BM LKS cells from flow cytometry (1 dot per mouse; average of 3 experiments shown; *n* = 8, 6, 5, 9, 9, 9, from left to right; mean ± SEM shown). (H) Quantification of BM LKS-SLAM (immunophenotypic HSCs) cells from flow cytometry (1 dot per mouse, numbers of mice as in G, mean ± SEM shown). *p* values were determined by two-tailed Mann-Whitney U test, ^∗^*p* < 0.05, ^∗∗^*p* < 0.01, ^∗∗∗^*p* < 0.001 ^∗∗∗∗^*p* < 0.0001. See also Figure S1.

We then asked whether this protection matters at the earliest stage of HSC development—during their emergence from the dorsal aortic endothelium starting at E10.5. Taking embryos at E10.5, we stained them for CD31, an endothelial marker, and c-Kit, a marker for emerging HSC clusters (Figure 2A).^36^^,^^37^^,^^38^ At this time point, *Adh5*^−/−^ *Fancd2*^−/−^ embryos exhibited a 4-fold reduction in hematopoietic clusters, compared with controls (Figures 2B and 2C). To firmly establish that formaldehyde protection is vital at this earliest stage of HSC development, we created a genetic model to test this. We engineered a conditional *Adh5* allele in which the critical exon (exon 3) is inverted (*Adh5*^*tm1a(switch)Kjpl*^, *Adh5*^*sw*^ hereafter), inactivating *Adh5* expression, and flanked by unidirectional *loxP* sites.^39^ At E11, Cre recombinase is expressed, driven by the blood-specific promoter *Vav1* (*Vav1-iCre*).^40^ This recombinase acts on *loxP* sites flanking the *Adh5* exon to revert it, thus restoring *Adh5* expression in blood during HSC emergence (Figure 2D). Thus, *Adh5*^*sw/−*^ *Vav1-iCre* mice are proficient in ADH5 in blood and deficient in ADH5 in the rest of the body (Figures 2E and S1D). *Adh5*^*sw/−*^ *Fancd2*^−/−^ *Vav1-iCre* mice show a substantial reduction in immunophenotypic LKS (Lin^−^ c-Kit^+^ Sca-1^+^) cells and HSCs (LKS-SLAM) in both the adult BM and fetal liver, compared with allelic controls (in adult BM, 11-fold reduction compared with WT and 5-fold compared with *Fancd2*^−/−^; in fetal liver, 4-fold reduction compared with *Adh5*^*sw/−*^ *Vav1-iCre* and equal to *Adh5*^−/−^ *Fancd2*^−/−^) (Figures 2F–2H and S1E). Thus, a lack of ADH5 during the time frame of HSC emergence has a profound effect on this critical process. Protection against endogenous formaldehyde is therefore crucial when HSCs emerge at the endothelial-to-hematopoietic transition in the dorsal aorta.

### Blood-specific ADH5 ablation reveals ongoing need for formaldehyde clearance

**Figure 3:**
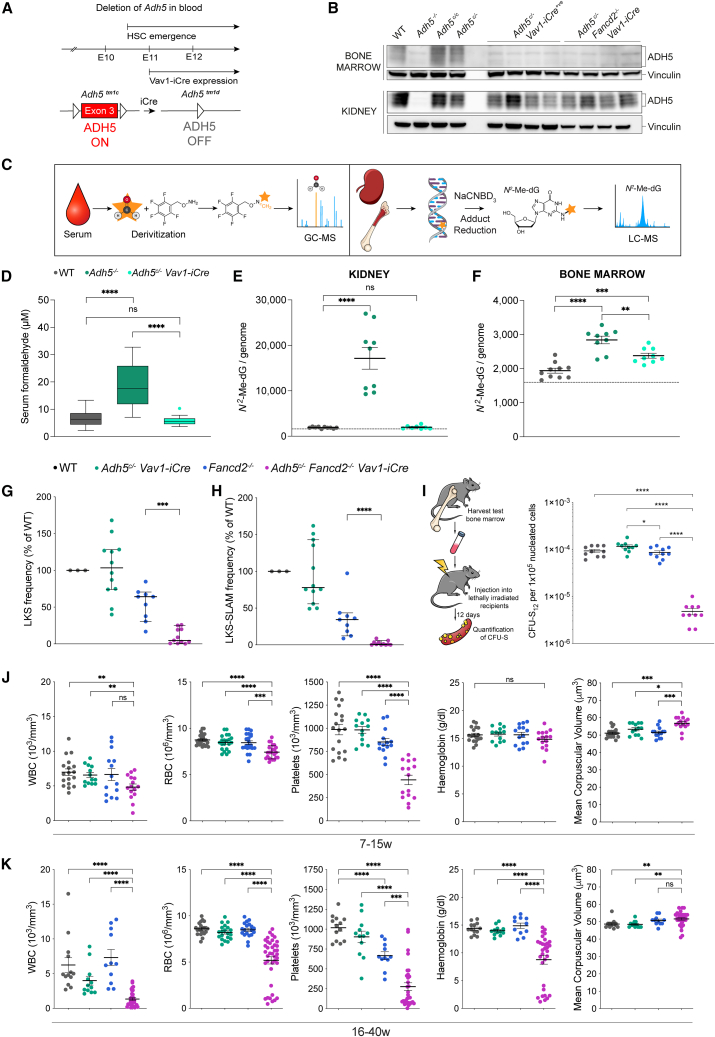
Hematopoietic-restricted ADH5 ablation reveals ongoing need for formaldehyde clearance (A) Generation of *Adh5*^*c/−*^*Vav1-iCre* mice, using the functional, conditional EUCOMM *Adh5*^*tm1c*^ floxed exon 3, excised by iCre from E11 onward to produce the non-functional *Adh5*^*tm1d*^ allele in blood. (B) Western blot of mouse *Adh5*^*c/−*^*Vav1-iCre* and *Adh5*^*c/−*^*Fancd2*^−/−^*Vav1-iCre* BM from individual mice demonstrating hematopoietic ADH5 deficiency and ADH5-proficient kidney, with vinculin as loading control. (C) Experimental outline by GC-MS quantitation of serum formaldehyde and the detection of the reduced formaldehyde-DNA adduct (*N*^2^-Me-dG) by LC-MS in tissues. (D) Serum formaldehyde levels in mice (*n* = 28, 15, 15, left to right). Quartiles and median shown by box; Tukey whiskers span 1.5 interquartile ranges. (E) LC-MS quantification of *N*^2^-Me-dG in kidneys. Dotted line indicates limit of detection (1 dot per mouse; *n* = 10, 9, 8, left to right; mean ± SEM shown). (F) LC-MS quantification *N*^2^-Me-dG in BM. Dotted line indicates limit of detection (1 dot per mouse; *n* = 10, 9, 9, left to right; mean ± SEM shown). (G) Flow cytometry quantification of BM LKS (immunophenotypic HSPCs) (1 dot per mouse; *n* = 15, 12, 9, 11, normalized to WT average within each of 3 experiments, left to right; mean ± SEM shown). (H) Flow cytometry quantification of BM LKS-SLAM (immunophenotypic HSC) cells (1 dot per mouse; numbers of mice as in G, left to right; mean ± SEM shown). (I) Outline of CFU-S assay, a quantitative proxy for short-term HSCs, and graphed results (1 dot per recipient mouse; *n* = 10, 10, 10, 10, left to right; mean ± SEM shown). (J) Blood counts of white blood cells (WBCs), red blood cells (RBCs), and platelets along with hemoglobin and mean corpuscular volume (MCV) from 7- to 15-week-old mice (1 dot per mouse; *n*_WT_ = 18, *n*_*Adh5 Vav1-iCre*_ = 13, *n*_*Fancd2*_ = 14, *n*_*Adh5 Fancd2 Vav1-iCr*e_ = 15; mean ± SEM). (K) Blood counts and indices from *Adh5*^*c/−*^*Fancd2*^−/−^*Vav1-iCre* and control mice (aged 16–40 weeks). Dotted horizontal line indicates lower end of normal range. (1 dot per mouse; *n*_WT_ = 13, *n*_*Adh5 Vav1-iCre*_ = 12, *n*_*Fancd2*_ = 11, *n*_*Adh5 Fancd2 Vav1-iCr*e_ = 31; mean ± SEM). *p* values were determined by two-tailed Mann-Whitney U test, ^∗^*p* < 0.05, ^∗∗^*p* < 0.01, ^∗∗∗^*p* < 0.001 ^∗∗∗∗^*p* < 0.0001. See also Figure S1.

It is unclear if formaldehyde protection in the blood compartment must be maintained throughout development and post-natal life. We therefore developed a conditional mouse model wherein floxed exon 3 of *Adh5* is deleted when *Vav1-iCre* is expressed (*Adh5*^*EUCOMM(tm1c)Wtsi*^, *Adh5*^*c*^ hereafter) (Figure 3A). This inactivates formaldehyde detoxification in the hematopoietic compartment starting from E11. This allele functions as predicted, with loss of the ADH5 protein in hematopoietic cells but not in the rest of the animal (Figures 3B, S1F, and S1G). We measured blood formaldehyde levels using derivatization followed by gas chromatography-mass spectrometry (GC-MS) (Figure 3C), which shows a reduction to WT levels (6.8 μM) in *Adh5*^*c/−*^ *Vav1-iCre* mice (Figure 3D). In addition, we assessed the levels of the formaldehyde adduct, *N*^2^-hydroxymethyl-deoxyguanosine (*N*^2^-Me-dG), using liquid chromatography-mass spectrometry (LC-MS), on DNA from kidney and BM of *Adh5*^*c/−*^ *Vav1-iCre* mice and controls. This shows that *N*^2^-Me-dG adducts on DNA from ADH5-proficient kidneys are equivalent to WT, as expected, while in the BM where ADH5 is absent, adduct levels were significantly raised above those of WT (Figures 3E and 3F). In accordance with adduct levels, using the peripheral blood micronucleus assay as a readout for DNA damage, we see that *Adh5*^*c/−*^ *Fancd2*^−/−^ *Vav-iCre* mice carry an intermediate burden of micronuclei, compared with *Fancd2*^−/−^ and *Adh5*^−/−^ *Fancd2*^−/−^ mice (Figure S1H). Altogether, these data suggest that although the serum formaldehyde levels are unchanged from WT in the *Adh5*^*c/−*^ *Fancd2*^−/−^ *Vav-iCre* mice, endogenous formaldehyde within blood cells triggers elevated DNA damage. Next, we assessed hematopoiesis in this model by flow cytometry. This showed a striking reduction in LKS and immunophenotypic HSC (LKS-SLAM) numbers in the adult and juvenile BM and fetal liver of *Adh5*^*c/−*^ *Fancd2*^−/−^ *Vav1-iCre* mice (75-fold fewer LKS in *Adh5*^*c/−*^ *Fancd2*^−/−^ *Vav1-iCre* adult BM, compared with WT; 3.4-fold fewer immunophenotypic HSCs in *Adh5*^*c/−*^ *Fancd2*^−/−^ *Vav1-iCre* fetal liver, compared with WT) (Figures 3G, 3H, and S1I–S1K). To assess BM regenerative capacity, we conducted a short-term spleen colony-forming unit (CFU-S) BM transplantation assay. This demonstrated that *Adh5*^*c/−*^ *Fancd2*^−/−^ *Vav1-iCre* short-term HSPCs had a profoundly reduced (19- to 35-fold compared with WT) ability to form splenic colonies (Figures 3I and S1l). Finally, we assessed blood production in 7- to 15-week-old and 16- to 40-week-old *Adh5*^*c/−*^ *Fancd2*^−/−^ *Vav1-iCre* mice. Unlike constitutive *Adh5*^−/−^ *Fancd2*^−/−^ mice, which die within 7 weeks, these animals have minor impairments in blood production up to a maximum age of 40 weeks, despite having very few HSPCs (Figures 3J and 3K). Altogether, although we see a marked attrition of HSPC numbers, severely reduced functional capacity of HSCs, and DNA damage within the blood compartment of *Adh5*^*c/−*^ *Fancd2*^−/−^ *Vav1-iCre* mice, blood production can be supported for several months.

### Sustained hematopoiesis despite profound HSC depletion

**Figure 4:**
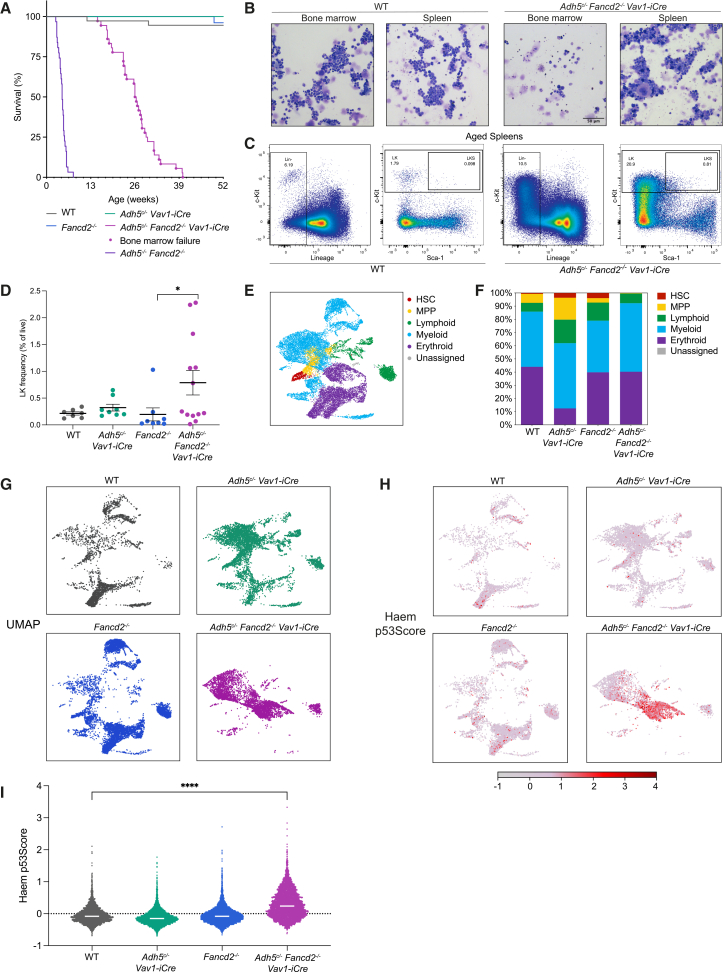
Sustained hematopoiesis in mice lacking blood-specific formaldehyde protection (A) Kaplan-Meier survival of *Adh5*^*c/−*^*Fancd2*^−/−^*Vav1-iCre* mice and genetic controls. Magenta dots on *Adh5*^*c/−*^*Fancd2*^−/−^*Vav1-iCre* represent deaths from BMF. *n*_*animals*_: WT = 38, *Adh5*^*c/−*^*Vav1-iCre* = 22, *Fancd2*^−/−^ = 27, *Adh5*^*c/−*^*Fancd2*^−/−^*Vav1-iCr*e = 36 (*Adh5*^*c/−*^*Fancd2*^−/−^*Vav1-iCr*e BMF deaths = 27). (B) Modified Wright-Giemsa-stained cytospins depict both reduced cellularity in BM and splenic hematopoiesis in aged *Adh5*^*c/−*^*Fancd2*^−/−^*Vav1-iCre* mouse, alongside WT control. Scale bar, 50 μm. (C) Representative flow cytometry plots of Lin^−^ c-Kit^+^ (LK) gating from aged spleens. (D) Flow cytometry proportions of LK gating from aged spleens in a subset of *Adh5*^*c/−*^*Fancd2*^−/−^*Vav1-iCre* mice (1 dot per mouse; *n* = 7, 8, 8, 11, from left to right; mean ± SEM). (E) Uniform manifold approximation and projection (UMAP) plot of single-cell 10× RNA-seq on LK cell transcriptomes from aged mouse spleens. All four genotypes superimposed; colors represent broad lineage clusters. (F) Proportions of cells from each broad lineage cluster from single-cell transcriptomic analysis in aged spleens. (G) UMAP visualization of LK transcriptomes from aged spleens, colored according to each genotype, shown separately to highlight differences in proportions of clusters. (H) UMAPs depicting Haem p53Score composed of 16 *Trp53* target genes in LK cells for each genotype, with light gray being negative for increased expression of these targets and dark red being highly positive for the target transcripts. (I) Quantification of Haem p53Score in each genotype by violin plot (*n* = 2,838, 5,689, 6,246, 4,104, left to right; median shown, Welch’s *t* test). *p* values were determined by two-tailed Mann-Whitney U test unless otherwise indicated; ^∗^*p* < 0.05, ^∗∗^*p* < 0.01, ^∗∗∗^*p* < 0.001 ^∗∗∗∗^*p* < 0.0001. See also Figure S2.

We monitored the health of *Adh5*^*c/−*^ *Fancd2*^−/−^ *Vav1-iCre* mice until they met humane endpoints. These animals live longer (median 26 weeks) than mice globally deficient in *Adh5* and *Fancd2* (median 4.6 weeks)^25^ (Figure 4A). Like the globally deficient mice, most *Adh5*^*c/−*^ *Fancd2*^−/−^ *Vav1-iCre* animals succumb to BMF (Figures S2A and S2B). However, the lifespan of and sustained blood production in these mice (up to 40 weeks) are striking, considering that they harbor near-undetectable levels of HSCs with minimal reconstitution capacity (Figures 3G–3I, 4B, S1I, S1J, and S1L). Splenic extramedullary hematopoiesis was noted in ∼40% of aged animals, and in this tissue, we could gate an immunophenotypic HSPC population (Figures 4B–4D). To interrogate the splenic hematopoietic potential using an orthogonal method, we performed single-cell RNA sequencing (scRNA-seq) on the splenic Lin^−^ c-Kit^+^ (LK) population of an aged mouse with splenic hematopoiesis, as compared with control mice (Figures 4E, 4F, S2H, and S2I). The aged *Adh5*^*c/−*^ *Fancd2*^−/−^ *Vav1-iCre* BM was too aplastic to carry out scRNA-seq (Figure S2C), unlike their younger adult counterparts (Figures S2D–S2G). Very few cells carrying an HSC expression profile were detected in aged WT spleen but were increased in both aged single knockout controls (Figure 4F). In the *Adh5*^*c/−*^ *Fancd2*^−/−^ *Vav1-iCre* mouse, however, there was no expansion of this putative HSC population, and additionally, there was a contraction in the multipotent progenitor (MPP) population (Figures 4F, 4G, and S2I). We profiled these populations for activation of p53 response by means of the Haem p53Score^41^ (Figures 4H and 4I). This showed a marked activation of the p53 response in *Adh5*^*c/−*^ *Fancd2*^−/−^ *Vav1-iCre* cells carrying profiles for the erythroid and myeloid lineages. In summary, removing formaldehyde protection in the hematopoietic compartment allows blood production to be sustained for many months, but over time, declining blood function can result in extramedullary hematopoiesis. While the spleen can act as a displaced site for hematopoiesis, it does not appear to provide a sanctuary to shield HSCs lacking formaldehyde protection.

### A mathematical modeling approach exploiting somatic variants reveals formaldehyde-induced CH

**Figure 5:**
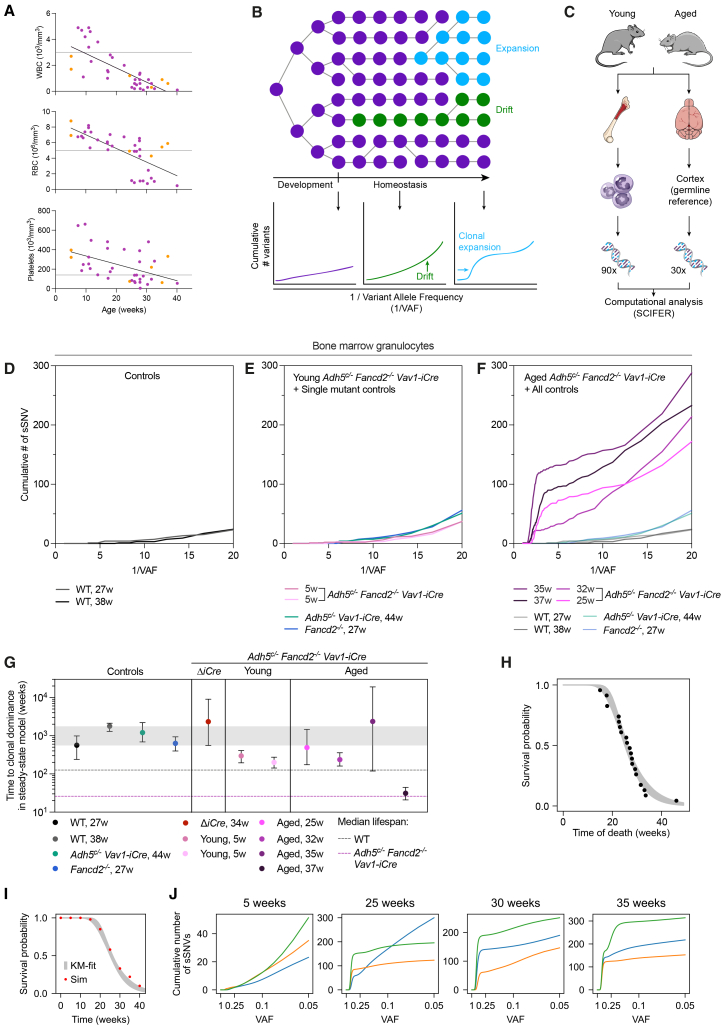
Eventual blood production from a single ancestor in mice lacking blood-specific formaldehyde protection (A) Counts from *Adh5*^*c/−*^*Fancd2*^−/−^*Vav1-iCre* mice displayed relative to age for WBCs, RBCs, and platelets (top to bottom), with best fit line (each dot represents a mouse at the indicated age; orange data points are terminal blood draws from mice sequenced in D–F below). R^2^_WBC_ = 0.5611; R^2^_RBC_ = 0.5220; R^2^_PLT_ = 0.2541. (B) Schematic of blood-forming cells over the life course and examples of expected VAF distributions, sampled at different time points. During development, blood-forming cells expand to form the basis of the hematopoietic compartment, and the number of somatic variants is low (left-most graph). Then, within the homeostatic phase, there is a progressive increase in variants accrued as passenger mutations resulting in neutral genetic drift (middle graph). Driver mutations can occur that result in clonal expansion and occupation of a disproportionate share of the population, reflected in a steep shoulder on the graph (right-most graph). For ease of displaying low VAFs, all graphs are shown as 1/VAF against the cumulative number of variants. (C) Experimental outline for SCIFER in mice. (D) Cumulative number of sSNVs and their frequency levels as detected in bulk (90×) WGS of BM granulocytes from aged WT genetic control mice, demonstrating polyclonal hematopoiesis. (E) Cumulative number of sSNVs and their frequency levels as detected by bulk (90×) WGS of BM granulocytes from young *Adh5*^*c/−*^ Fancd2^−/−^*Vav1-iCre* mice and aged *Adh5*^*c/−*^*Vav1-iCre* and *Fancd2*^−/−^ genetic controls, demonstrating polyclonal hematopoiesis. (F) Cumulative number of sSNVs and their frequency levels as detected by bulk (90×) WGS of BM granulocytes from 4 aged *Adh5*^*c/−*^*Fancd2*^−/−^*Vav1-iCre* mice, demonstrating that progression to CH has occurred (apparent as steep shoulder). Aged genetic controls shown for reference. (G) Predicted time to clonal dominance, using the steady-state model: in genetic controls, the *ΔiCre* mouse that lost *Vav1-iCre*, preventing *Adh5* deletion (highlighted by vertical line separators), and *Adh5*^*c/−*^*Fancd2*^−/−^*Vav1-iCre* young and aged mice. Gray-shaded bar indicates time interval when WT mice could reach clonal dominance. Dotted lines represent median survival. Error bars are 95% confidence intervals. (H) Modeling of BFA attrition. This attrition model is based on time of death of *Adh5*^*c/−*^*Fancd2*^−/−^*Vav1-iCre* mice whose eventual demise was a result of BMF (shown here as black dots; same data as in Figure 4A). Gray shading shows model fit. Within the model, BFA loss equates with death of mice. BFA attrition is modeled as a stochastic birth-death process (with birth describing self-renewing divisions and death being cell death, differentiation, or senescence). For an initial, post-natal number of BFAs of ∼100, the model predicts a mean survival time of an BFA of 3 weeks and a probability of 35%–40% that a BFA divides into two BFAs before being lost. (I) Numerical simulations (red) follow the estimated survival probabilities (gray), based on the attrition model. (J) Using the attrition model, 100 mice were simulated and the cumulative number of sSNVs versus VAF were computed at different time points. Shown are three representative examples for each indicated time point. See also Figures S3, S4, and S5.

Blood production can be sustained for many months in *Adh5*^*c/−*^ *Fancd2*^−/−^ *Vav1-iCre* mice, despite their possessing very few functional HSCs. This contrasts with *Adh5*^−/−^ *Fancd2*^−/−^ mice that, on average, succumb to BMF within a month of birth^25^ (Figure 4A). While *Adh5*^*c/−*^ *Fancd2*^−/−^ *Vav1-iCre* animals survive longer, they do show a progressive decline across the output of the three major blood constituents (Figures 3J, 3K, and 5A). In most animals, this culminates in BMF (87% of deaths) at an average age of 26 weeks (Figures 4A, S2A, and S2B). A key question is how these mice still manage to produce blood for so long. To address this, we used a new computational inference method known as selected clone inference (SCIFER), which is based on the variant allele frequency (VAF) distribution of somatic single-nucleotide variants (sSNVs) present within a sampled population.^20^ The principle of SCIFER and how it enables us to infer whether a population might be shaped by neutral drift or clonal expansion are illustrated in Figure 5B. We extracted granulocytic genomic DNA for deep WGS (90×) from *Adh5*^*c/−*^ *Fancd2*^−/−^ *Vav1-iCre* and control mice. In the same animals, we also sequenced brain DNA (30×) to provide a germline reference, enabling us to curate granulocytic-specific sSNVs (Figure 5C). We chose BM granulocytes because of their very short lifespan (3–5 days), affording us the crucial ability to obtain a snapshot of the current originators of hematopoiesis. Although SCIFER infers information about the population dynamics of ancestors of detectable clones from which the blood population arises (Table S1), it does not indicate if these entities are necessarily immunophenotypic HSCs; we therefore call them blood-forming ancestors (BFAs).

Applying SCIFER to the VAF data of sSNVs present in granulocytes taken from aged WT (28–38 weeks), *Adh5*^*c/−*^ *Vav1-iCre* (44 weeks), and *Fancd2*^−/−^ (27 weeks) mice, we see that these produced blood in a polyclonal manner and fit a neutral drift model (Figures 5B, 5D, 5E, and S3A). Additionally, from analysis of WGS data from the young *Adh5*^*c/−*^ *Fancd2*^−/−^ *Vav1-iCre* mice, SCIFER estimates several thousand BFAs at birth for both mice (Tables S1 and S3B), and again, in these mice, we see the best fit is one of neutral drift (Figures 5B, 5E, and S3B). This indicates that the granulocyte population in these young animals arises from a polyclonal BFA population, explaining how blood production is robustly sustained at this point. It is noteworthy, however, that young *Adh5*^*c/−*^ *Fancd2*^−/−^ *Vav1-iCre* mice already accrue an sSNV profile comparable to that of the aged controls, indicative of an increased mutational burden (Figure 5E). In aged *Adh5*^*c/−*^ *Fancd2*^−/−^ *Vav1-iCre* mice (25–37 weeks), the picture dramatically shifts. All four animals exhibit elevated sSNV levels, VAF distributions that fit a clonal expansion model, and a predicted clone possessing a VAF of approximately 0.5 in the population, indicating every cell had one copy of the variant allele; thus, current blood production traces back to a single BFA in these aged animals, contrasting sharply with polyclonal granulocyte production in young *Adh5*^*c/−*^ *Fancd2*^−/−^ *Vav1-iCre* animals (Figures 5F and S3C; Tables S1 and S2). While the burden of mutations was higher in *Adh5*^*c/−*^ *Fancd2*^−/−^ *Vav1-iCre* mice, particularly in the aged animals (Figures S3D–S3G), their genomic positioning showed the same broad distributions as control mice (Figure S3H). Importantly, variants occurring at a VAF of 0.5 in granulocytes of each mouse were only partly present in their respective splenic B cell populations, indicating that these clones were not a result of an early mutation in the mesoderm embryonic germ layer (the cortex germline reference derives from ectoderm) (Figure S3J). This also demonstrates that in B cells, which are a long-lived population and therefore offer a historical account of hematopoietic production, *Adh5*^*c/−*^ *Fancd2*^−/−^ *Vav1-iCre* animals did produce blood from a polyclonal pool of BFAs (Figure S3J). Altogether, these data point to a role for progressively declining BFA diversity in the eventual development of CH.

The timing of the origin for the clonal BFA in the aged *Adh5*^*c/−*^ *Fancd2*^−/−^ *Vav1-iCre* animals can be estimated from the accrued clonal sSNV total. While there was variation seen in the cumulative sSNV count in the dominant clone of each aged *Adh5*^*c/−*^ *Fancd2*^−/−^ *Vav1-iCre* animal (from 20 to 120), based on known mutation acquisitions in HSPC (∼50 accrued to the point of birth),^21^ clone emergence would be placed before or just after birth (Table S1). Additionally, SCIFER allows the extraction of expected time to fixation of a clone by neutral drift in a homeostatic BFA pool (given by the ratio of BFA number to BFA division rate) (Figure 5G). For WT controls, the time it would take to reach complete clonal dominance by neutral drift was inferred to be in the order of 1,000 weeks (equivalent to ∼20 years) and hence well beyond a mouse’s lifetime (Figure 5G; gray-shaded bar highlights the time interval when WT mice may reach clonal dominance). This is consistent with the notion that selection is required for CH to develop.^21^ By contrast, in young *Adh5*^*c/−*^ *Fancd2*^−/−^ *Vav1-iCre* mice, SCIFER inferred markedly accelerated neutral drift, on the order of 100 weeks (Figure 5G; the variable inferences for expected time to clonal fixation in the aged *Adh5*^*c/−*^ *Fancd2*^−/−^ *Vav1-iCre* mice characterize the sub-clonal dynamics within the dominant BFA clone and hence are not informative on how the BFA clone emerged in the first place). While this is still larger than the typical lifetime of these mice, the strong drop in the neutral drift timescale suggests that neutral dynamics could contribute to the establishment of clonal granulocyte production.

Hence, we queried how the emergence of the dominant clones in the *Adh5*^*c/−*^ *Fancd2*^−/−^ *Vav1-iCre* mice from a polyclonal population of BFAs can be explained. Since most mice die of BMF, we asked whether neutral drift in a progressively declining population of BFAs could explain both CH emergence in individual aged animals (Figure 5F) and the Kaplan-Meier curve of the *Adh5*^*c/−*^ *Fancd2*^−/−^ *Vav1-iCre* cohort (Figure 4A). We modeled BFA attrition as a stochastic birth-death process (with “birth” describing self-renewing division of one BFA into two BFAs and “death” being loss of a BFA). To describe BFA attrition, we let the loss rate exceed the self-renewal rate. Hence, BFAs become extinct in a stochastic manner, causing their complete loss after variable times in individual mice. For simplicity, we equate complete BFA loss in the model with BMF and death of the mouse. This modeling quantitatively accounts for the Kaplan-Meier curve (Figure 5H, Kaplan-Meier data from experimental mice; Figure 5I, from simulated mice). For an initial (post-natal) number of BFAs of ∼100 (corresponding to ∼1% of normal HSC number^21^), the model predicts a mean survival time of a BFA of 3 weeks and a probability of 35%–40% that a BFA divides into two BFAs before being lost (Figures 5J, S4A, and S4B). Importantly, hematopoiesis in long-surviving simulated mice becomes clonal, as is seen in the experimental animals (Figure 5J compared with Figure 5F). Thus, a model of neutral drift in the context of stochastic attrition of BFAs can account for both the development of CH in *Adh5*^*c/−*^ *Fancd2*^−/−^ *Vav-iCre* mice and their overall survival statistics without a requirement for positive selection.

Genomic analysis predicted a translocation between chromosomes 13 and 16 in about 70% of the granulocytic population in one aged *Adh5*^*c/−*^ *Fancd2*^−/−^ *Vav1-iCre* animal (Figure S5A). This allowed for an opportunity to validate the genomics pipeline used for SCIFER by cytogenetics using multicolor fluorescence *in situ* hybridization (M-FISH). This revealed that 33% of metaphases carried t(13;16), which is broadly consistent with granulocyte proportions in the BM preparation as assessed by fluorescence-activated cell sorting (FACS) (11%) (Figure S5B). Additionally, in a single *Adh5*^*c/−*^ *Fancd2*^−/−^ *Vav1-iCre* mouse, deep WGS revealed that the granulocyte population arose from a single BFA that had lost the *Vav1-iCre* transgene (Figure S5C). Whereas most mice have complete excision of *Adh5* by iCre in blood, this clone escaped ADH5 deletion, and hence, this animal was partially proficient in formaldehyde protection (and is hereafter referred to as *ΔiCre*) (Figure S5D). The founding BFA in the *ΔiCr*e mouse had 25 sSNVs (Figure S5E), suggesting that it likely arose in a time frame concurrent with the embryonic onset of *Vav1-iCre* expression.^40^^,^^42^ Reassuringly, the estimated time to fixation by neutral drift for this *ΔiCre* animal was within the same range as that of WT mice (enclosed red data point shown in Figure 5G), suggesting that the BFA clone emerging from the embryonically selected cell in this *ΔiCre* mouse displayed similar dynamics to that of the BFA population in a WT mouse.

Monoclonal blood production seen in aged *Adh5*^*c/−*^ *Fancd2*^−/−^ *Vav1-iCre* animals could have arisen by selection (Figures 5B and 5F). We therefore looked at whether potential drivers associated with clonal expansion were present in more than one aged *Adh5*^*c/−*^ *Fancd2*^−/−^ *Vav1-iCre* mouse. Only one gene, *Eef1a1* (not involved in CH), had variants in more than one aged mouse, but their VAF frequencies were too low to play any role in clonality (Table S3). Curation of all genes (impacted by exonic sSNVs, SVs, insertion or deletion [indels], and CNAs) in all aged mice did not reveal any currently known genetic routes to human CH, nor did they fall into common pathways involved in that phenomenon (STAR Methods; Table S3). We additionally compared the curated gene list from aged animals with the genes affected by sSNVs reported recently in mouse hematopoiesis and in the report of an 115-year-old individual who had oligoclonal blood production.^13^^,^^21^ No shared genes were seen that are known to play a role in driving CH. In summary, there was an absence of known drivers in the clones of *Adh5*^*c/−*^ *Fancd2*^−/−^ *Vav1-iCre* animals.

While SCIFER modeling can reveal blood production sustained by a single BFA in the near absence of experimentally validated HSCs in the *Adh5*^*c/−*^ *Fancd2*^−/−^ *Vav1-iCre* model, what remains unclear is the cause of the eventual decline to BMF in these animals. We reasoned that with a severely reduced number of cells supporting blood production, replicative shortening of telomeres^43^^,^^44^ could be responsible. To assess this, we performed telomere FISH on nuclei from BM of two mice with BMF, as well as two littermate WT controls. To set up a custom telomere analysis pipeline, telomeric FISH was performed on WT mice alongside a mouse strain known to have short telomeres (*Mus spretus*),^45^^,^^46^ which shows that intensity measures made on short *Mus spretus* telomeres reveal a markedly different range of intensities when compared with WT (Figures S5F and S5G). Telomeric FISH intensities from *Adh5*^*c/−*^ *Fancd2*^−/−^ *Vav1-iCre* animals (both in BM crisis) versus WT animals were then assessed; these did not show consistent differences in their telomere lengths (Figure S5H). Telomere shortening is therefore not an explanation for the eventual decline to BMF in this model.

### SCIFER detects oligoclonal hematopoiesis in a child with FA

**Figure 6:**
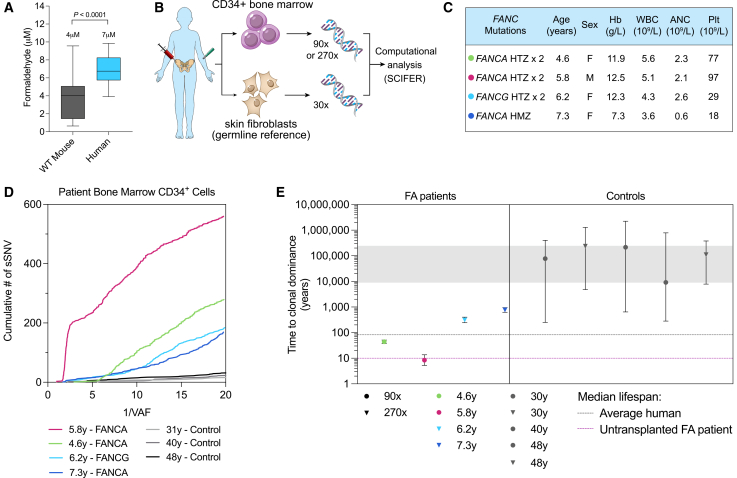
Emergence of clonal blood production in an FA patient (A) Serum formaldehyde levels in WT mice and humans by GC-MS (*n* = 29, 26, left to right). Quartiles and median shown by box; Tukey whiskers span 1.5 interquartile ranges; two-tailed Mann-Whitney *U* test. (B) Experimental outline for SCIFER analysis in FA patients. Samples for SCIFER were taken at the time of diagnosis, where patients exhibited mild BM dysfunction. (C) Summary of patient FA complementation group mutations, age, sex. Hemoglobin level, WBC count, absolute neutrophil count, and platelet count are those at time of experimental sample collection. HTZ, heterozygous; HMZ, homozygous. (D) Cumulative number of sSNVs and their frequency levels as detected by bulk WGS result from patient BM CD34^+^ cells. (E) Time to reach clonal dominance in FA children versus healthy human controls. Gray-shaded bar indicates the time interval when an unaffected individual could reach clonal dominance. Dotted lines are median lifespans for average humans and untransplanted FA patients. Error bars are 95% confidence intervals. See also Figure S6.

Seeing the striking CH resulting from attrition in mice, we next asked whether oligoclonal hematopoiesis occurs in humans. Humans lacking tier 1 protection against formaldehyde are very rare and display a severe phenotype akin to FA.^47^^,^^48^ In mice that only lack FA repair, the hematopoietic phenotype is discreet, unlike their human counterparts.^49^ We postulate this is due to increased formaldehyde clearance in mice compared with humans, which could reduce the DNA damage burden in these animals. This could explain their high tolerance to methanol, a formaldehyde precursor.^28^ We therefore determined blood formaldehyde levels in humans, which showed that they have higher levels than those seen in mice (Figure 6A). We then assessed hematopoiesis using SCIFER in four pediatric FA patients, who had been diagnosed with mild BM dysfunction and discreet cytopenia in their initial stages but did not have evidence of clonal, pre-leukemic disease (Figures 6B, 6C, and S6A). Upon deep WGS of CD34^+^ cells (at 90× or 270× depth) and application of SCIFER modeling to the data, the picture seen in these children is shown in Figures 6D and S6B. Three individuals show a fit explainable by drift (4.6-, 6.2-, and 7.3-year-old individuals), but the time to achieve clonal dominance by neutral drift is greatly shortened, compared with much older human controls (30–48 years of age) (Figure 6E). In one FA individual (age 5.8 years), blood production was supported by a single BFA estimated to have emerged at 3 years of age. This individual’s estimated time to clonal dominance by neutral drift was the lowest (Figure 6E; in the order of ∼10 years). Sequencing of the index *FANCA* mutations ruled out somatic reversion (data not shown).^50^ Investigation of the genes in which non-synonymous sSNVs were arising showed no evidence of CH driver mutations in this individual (Table S4). Taken together, these results point toward a mechanism for CH in FA patients arising from attrition of HSPCs.

## Discussion

Protection against formaldehyde is critical for preserving genomic integrity in HSCs, from their genesis through to post-natal life. Removing formaldehyde protection leads to profound HSPC depletion culminating in monoclonal CH, followed by a cessation in blood production. Formaldehyde accumulation in HSPCs results in increased mutation load and pattern, which show similarity to SBS5.^47^ Although the dominant sources of endogenous formaldehyde are currently unknown, possible sources include enzymatic demethylation of histones/nucleic acids and folic acid decomposition.^26^^,^^51^ It is plausible that demethylation that often accompanies cell transition states could in part explain the vulnerability of HSCs to formaldehyde.^52^ It will be important to establish the main sources of endogenous formaldehyde production.

**Figure 7:**
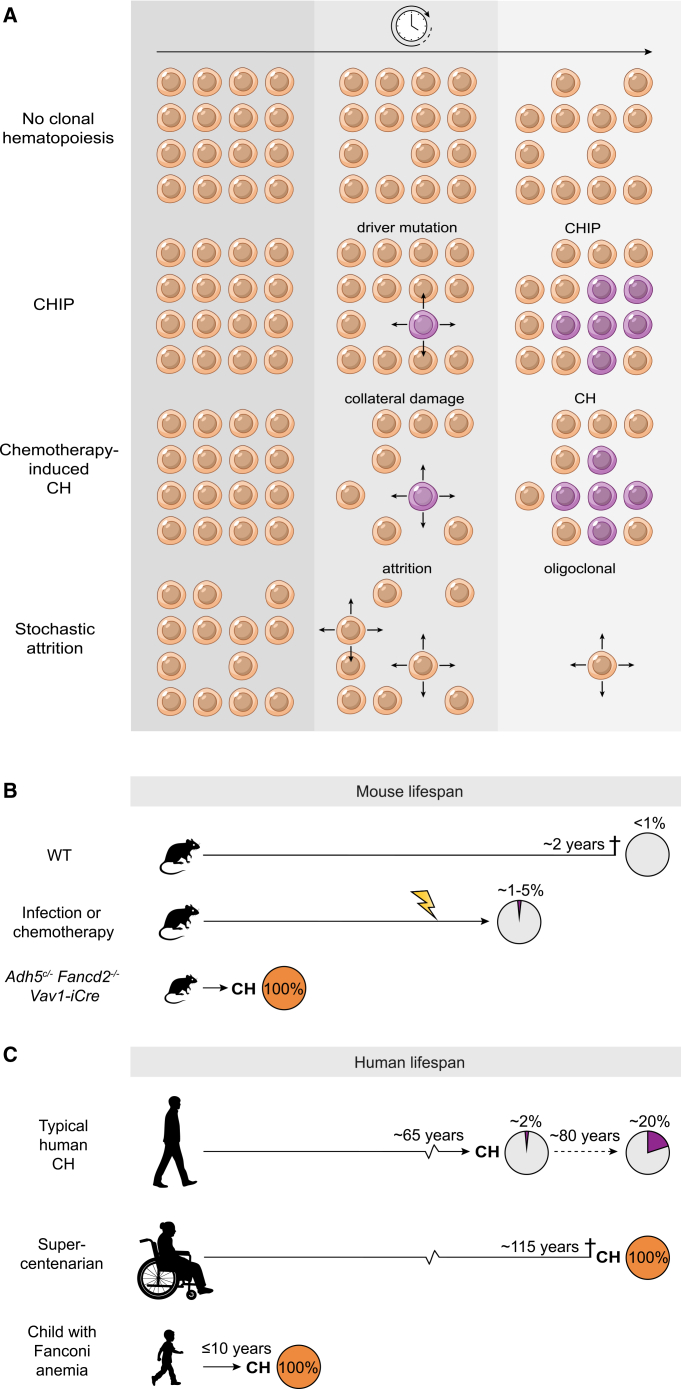
Models for CH development (A) Model showing four different modes of clone accumulation over time. In the case of no CH, the pool of functional blood-forming cells remains mostly stable. In the case of CHIP, a driver mutation may occur, and the clone may expand disproportionately over time to occupy a greater proportion of the blood-forming cells. Similarly, chemotherapy can favor CH, owing to survival and expansion of clones carrying advantageous mutations. Lastly, progressive stochastic attrition could result in oligoclonal hematopoiesis and, in extreme cases, culminate in monoclonal hematopoiesis. Purple represents cells with a driver mutation, gray represents dying or differentiating cells. (B) CH (hereafter depicted as a pie chart) is typically defined as having a clone comprising at least 2% of blood. In WT mice, CH does not occur within their natural lifespan. Infection or treatment with chemotherapy can induce the outgrowth of clones within mice, as reported by Kapadia et al.^21^ In contrast, *Adh5*^*c/−*^*Fancd2*^−/−^*Vav1-iCre* mice spontaneously develop monoclonal hematopoiesis within months. Orange represents events with no known driver mutations. (C) Humans are much longer-lived than mice. By the age of 65 years, at least 10% of people develop age-related CH, with at least 2% of blood being derived from a detectable clone. Both the clone size and the number of people affected tend to increase with time. In a 115-year-old individual, 100% of blood was found to be clonal, with no known driver mutations.^13^ We found a pediatric FA patient with mild BM dysfunction to have monoclonal hematopoiesis, exemplifying 25-fold accelerated aging, again with no known driver mutations. Orange represents events with no known driver mutations; purple represents those with a driver mutation.

To date, analysis of aged mice has failed to detect CH, unless they are challenged by chemotherapy or inflammatory stimuli, whereby CH appears as a small clone size in contrast to what we describe here.^21^^,^^53^ An important highlight of our study is the use of SCIFER to uncover how blood is still being produced in severely HSPC-depleted, but otherwise healthy, animals; canonical methods fail to capture how hematopoiesis was being sustained. Similarly, in three FA children, despite their BM dysfunction, SCIFER detected an appreciable diversity of BFAs supporting blood renewal. Remaining BFAs may be exposed to further DNA damage-induced attrition, such as from aldehyde crosslinks that impede replication and degrade into DNA strand breaks when not repaired.^54^^,^^55^ A key advantage of SCIFER is the ability to exploit the use of sSNVs as naturally occurring barcodes. This is particularly important in understanding clonality when HSC attrition or senescence, rather than positive selection, are the dominant forces for the emergence of CH (Figure 7A). We schematically represent how attrition-based CH might emerge over time; this contrasts with CH associated with driver mutations and chemotherapy-triggered CH—the latter being a balance of selection and attrition (Figures 7B–7D).

Pioneering studies have uncovered driver-associated CH emerging in recovering BM in genetic and immune-driven aplastic anemia.^56^^,^^57^^,^^58^^,^^59^ Likewise, deep genomic analysis of reconstituted hematopoiesis after BM transplantation also detects emergence of driver-associated CH in the recipient, which is not apparent in the donor; it is postulated that a “pruning” process of HSCs devoid of drivers enables such donor-derived CH to emerge in the transplant recipient.^60^ To date, studies of CH have focused on surviving clones and fitness-promoting variant alleles, but many CH studies show clonal emergence despite the absence of any known driver mutations.^12^ Furthermore, most studies utilize targeted and exome sequencing, which bias toward detecting clones with driver mutations. These approaches fail to capture clones with variants in intergenic and intronic regions.^7^^,^^8^^,^^14^^,^^22^^,^^61^^,^^62^^,^^63^ Some of these studies also lack a non-hematopoietic germline reference tissue, often requiring a higher VAF threshold to call a clone.^7^^,^^14^^,^^61^^,^^62^^,^^64^^,^^65^

CH lacking known driver mutations may arise for several reasons: some mutations may confer fitness advantages, which are not yet understood, and non-mutational drivers have also been proposed (epigenetic or mitochondrial fitness changes^66^). Perhaps, in this context, stochastic loss of HSPCs could explain CH that arises in driver-less contexts. A useful analogy is to consider a race that may be won by the fittest runner (clone with driver variant). In contrast, as postulated by Kimura on the fixation of polymorphisms in population genetics, other races may be won by average runners (clones with passenger variants) because the other contestants stochastically drop out—the odds increase as the population contracts.^67^^,^^68^ The concept of CH resulting from a diminished stem cell pool was first conjectured in the context of aplastic anemia and inferred again based on the results of lineage tracing in a variety of tissues.^69^^,^^70^^,^^71^ Here, we show that stochastic attrition can greatly accelerate neutral drift, supporting this conjecture and extending upon Kimura’s landmark hypothesis rooted in population genetics.

The most striking evidence for attrition as a pathway to CH comes from WGS on blood from a 115-year-old woman, uncovering bi-clonal hematopoiesis in the absence of driver mutations.^13^ The proportion of people with CH aged >80 is substantial and on a trajectory that suggests with enough time, CH is inevitable.^12^^,^^15^^,^^61^^,^^72^ Furthermore, although immunophenotypic “HSCs” may increase with age, the potency and number of functional, blood-regenerating cells diminish, which may underlie the causal basis of anemia in later life. Altogether, our study exemplifies that understanding the evolution of CH, with or without mutational drivers, will require a better understanding of the exposures and physiological assaults leading to the gradual depletion of the functional HSC population.

### Limitations of the study

The monoclonal hematopoiesis described in our study is a consequence of genetic perturbation where formaldehyde accumulation drives DNA damage and HSC attrition. While we demonstrate its impact, the metabolic sources and regulation of formaldehyde production remain unresolved. Additionally, we do not show how formaldehyde-induced damage shapes CH in humans and mice when both its clearance and repair of the damage it causes remain intact. The inference of stem cell dynamics by SCIFER assumes a single BFA population and does not consider the potential role of a hierarchy of stem cells and MPPs. Furthermore, only four FA patients were analyzed, restricting its generalizability. We find no drivers, but it is still plausible that a form of selection, whether epigenetic or mutational, could confer a selective advantage. The failure to detect any such event does not firmly exclude a selective component that could culminate in survival of a final, single clone (BFA); however, we emphasize that because of the eventual BMF in our models, this clone must perish.

## Resource availability

### Lead contact

Further information and requests for resources and reagents should be directed to the lead contact, Ketan J. Patel (ketan.patel@imm.ox.ac.uk).

### Materials availability

*Adh5*^*tm1a(switch)Kjpl*^, and *Adh5*^*tm1c*^ and *Adh5*^*tm1d*^ mice (derived from *Adh5*^*tm1a*^ allele (C57BL/6N-*Adh5*^*tm1a(EUCOMM)WtsiH*^) are available from the lead contact upon request.

### Data and code availability

• scRNA-seq datasets have been deposited in the National Center for Biotechnology Information Gene Expression Omnibus repository as GEO: GSE316966 and are publicly available as of the date of publication.
• Murine sequencing data used for SCIFER analysis have been deposited at ENA: PRJEB107797 and are publicly available as of the date of publication. In accordance with French law, de-identified patient genomics data can be made available upon scientifically motivated interest; requests should be made through DRIVE (Direction Recherche, Innovation, Valorisation et Ecoles doctorales), University Paris-Cite (direction.drive@u-paris.fr). Any additional data reported or required to reanalyze data reported in this paper will be shared by the lead contact upon request.

## Acknowledgments

We thank the following: K.J.P. laboratory members for critical reading of the manuscript; MRC WIMM Flow Cytometry, Imaging facilities and University of Oxford BMS for technical help; members of the M.F.T.R.d.B. lab for assistance with embryo imaging; J. Hamley and David Sims, MRC WIMM for genomic analysis; Ares Animal Facility Cambridge and Cambridge Institute for Medical Research Flow Cytometry facility; L. Pytowski at Pixel Biology Ltd. for interphase telomere FISH analysis script development; and members of P. Vyas’s lab for advice on material preparation for SCIFER. This work was supported by the Medical Research Council (MRC) MC_UU_00029 to K.J.P. and MFTRB (A.N.K.-L. and J.M.B.); NC3R Training Grant NC/X00158X/1 to M.N.; Rubicon Grant from NWO (2020/38703/ZONMW) to E.G.; Wellcome Trust (106202/Z/14/Z) to K.J.P. (C.L.M. and F.L.); CRUK to K.J.P. (F.A.D.) (A23273) and M.W. (C60150/A23919); The Hubrecht Institute to J.I.G.; DKFZ-MOST Collaboration program, Project Ca 211, and Deutsche Forschungsgemeinschaft (DFG) (497777320) to T.H. (M.G., V.K., and N.C.); Association Française pour la Maladie de Fanconi (AFMF), the Fédération Leucémie Espoir, the Centre de Référence Aplasie Médullaire – RIME, Saint-Louis Robert Debré, and the IHU ANR program to the Paris Saint-Louis Leukemia Institute to J.S.; Wellcome Trust (206328/Z/17/Z, 203151/Z/16/Z, 203151/A/16/Z, and 226795/Z/22/Z), MRC (MC_PC_17230 and MR/S036113/1), Blood Cancer UK (18002), CRUK (C1163/A21762), and Aging Biology Foundation to B.G. (N.K.W. and T.I.); and Funai Foundation for Information Technology and the Honjo International Scholarship Foundation to T.I. Further support came from grants to the MRC WIMM Flow Cytometry Facility (MRC [MC_UU_12010 and MC_UU_12009], NIHR Oxford BRC, Kay Kendall Leukaemia Fund [KKL1057], John Fell Fund [131/030 and 101/517], the EPA fund [CF182 and CF170] and the MRC WIMM Strategic Alliance [G0902418 and MC_UU_12025]) and Wolfson Imaging Centre Oxford (MRC WIMM Strategic Alliance [G0902418], MRC [MC_UU_12009 and MC_UU_12010], Wolfson Foundation [18272], and Wellcome Trust [Micron 107457/Z/15Z]).

## Author contributions

Conceptualization, K.J.P., A.N.K.-L., and J.I.G.; methodology, J.M.B., M.G., F.E., C.L.M., E.G., M.N., and V.K.; investigation, A.N.K.-L., J.M.B., M.G., F.E., L.L., H.R., F.L., T.I., N.K.W., F.A.D., R.L.C., M.W., C.L.M., N.C., and J.I.G.; writing—original draft, K.J.P., T.H., A.N.K.-L., J.M.B., and M.G.; writing—review and editing, K.J.P., T.H., A.N.K.-L., J.M.B., M.G., V.K., and C.L.M.; funding acquisition, K.J.P., M.N., E.G., M.W., B.G., M.F.T.R.d.B., T.H., and J.S.; resources, K.J.P.; and supervision, K.J.P., T.H., B.G., M.F.T.R.d.B., and J.S.

## Declaration of interests

The authors declare no competing interests.

## Declaration of generative AI and AI-assisted technologies in the writing process

The authors used ChatGPT Edu as a starting point for the highlights and eTOC blurb. The authors reviewed, amended, and edited this and accept full responsibility for the final content.

## STAR★Methods

### Key resources table

**Table:** 

REAGENT or RESOURCE	SOURCE	IDENTIFIER
**Antibodies**		

goat anti-CD31	R&D Systems	Cat# AF3628; RRID:AB_2161028
rat anti-C-KIT	eBioscience	Cat# 14-1171-85 RRID:AB_467434
CD3e 145-2C11 FITC	eBioscience	Cat# 35-0031-U500 RRID:AB_2621659
CD4 H129.19 FITC	BD	Cat# 130308 RRID:AB_1279237
CD8a 53-6.7 FITC	BD	Cat# 553031 RRID:AB_394569
Mac-1 M1/70 FITC	BD	Cat# 553310 RRID:AB_394774
CD11c FITC	eBioscience	Cat# 11-0114-85 RRID:AB_464941
B220 RA3-6B2 FITC	BD	Cat# 553088 RRID:AB_394618
FceR1a MAR-1 FITC	eBioscience	Cat# 11-5898-85 RRID:AB_465309
Gr-1 (Ly-6G) RB6-8C5 FITC	BD	Cat# 553127 RRID:AB_394643
Ter119 FITC	BD	Cat# 116206 RRID:AB_313707
CD41 FITC	BD	Cat# 553848 RRID:AB_395085
c-kit (CD117) APC eF780	eBioscience	Cat# 47-1171-82 RRID:AB_1272177
Sca-1 PE-Cy7 clone D7	eBioscience	Cat# 25-5981-82 RRID:AB_469669
CD150 BV785	BioLegend	Cat# 11593
CD48 Biotin	BioLegend	Cat# 103410 RRID:AB_528827
Flt3 (CD135) PE A2F10	eBioscience	Cat# 12-1351-82 RRID:AB_465859
CD34 AF700	eBioscience	Cat# 56-0341-82 RRID:AB_493998
CD16_32 (FcgRII/III) APC	eBioscience	Cat# 17-0161-82 RRID:AB_469356
IL7Ra (CD127) BV605	BioLegend	Cat# 135025 RRID:AB_2562114
CD4 BV421 Clone H129.19	BD	Cat# 740024
CD8a BV786 Clone 53_6.7	BD	Cat# 563332 RRID:AB_2721167
Gr1/Ly-6G FITC RB6-8C5	Bioscience	Cat# 553127 RRID:AB_394643
B220/CD45R APC Clone RA3-6B2	BD	Cat# 553092 RRID:AB_398531
CD3e BV605 Clone 145-2C11	BD	Cat# 563004 RRID:AB_2737945
Ter119 PE-Cy7 Clone Ter119	BD	Cat# 55785
IgM APCef780 Clone II/41	eBioscience	Cat# 47-5790-82 RRID:AB_2573984
CD71 PE Clone R17 217.1.4	eBioscience	Cat# 12-0711-82 RRID:AB_465740
Mac/CD11b BV711 M1/70	BD	Cat# 563168 RRID:AB_2716860
B220 PerCP-Cy5.5	BioLegend	Cat# 103236 RRID:AB_893354
CD11b PE	BD	Cat# 553311 RRID:AB_394775
Gr-1 PE	BioLegend	Cat# 108407 RRID:AB_313372
Ter119 PE-Cy7	Invitrogen	Cat# 25-5921-82 RRID:AB_469661
CD45.1 BV421	BioLegend	Cat# 110732 RRID:AB_2562563
CD45.2 APC	BioLegend	Cat# 109814 RRID:AB_389211
Rabbit anti ADH5 clone 48	N/A	N/A
Mouse anti vinculin clone V284	EMD Millipore	Cat# 05-386 RRID:AB_11212640
anti-CD71 FITC, clone R17217.1.4	eBioscience	Cat# 11-0711-82 RRID:AB_465124
Donkey anti-Rat IgG (H+L) Alexa Fluor™ 568	Invitrogen	Cat# A78946 RRID:AB_2910653
Streptavidin BV421	BioLegend	Cat# 405225
Alexa 488 donkey anti-goat IgG		Cat# A1105
Swine anti-rabbit immunoglobulins HRP	Dako	Cat# P0217 RRID:AB_2728719
goat anti-mouse HRP	Invitrogen	Cat# 31432

**Chemicals, peptides, and recombinant proteins**		

BSA	Sigma Aldrich	Cat# A4503
7-AAD	BioLegend	Cat# 420404
HindIII-HF	New England Biolabs	Cat# R3104S
Bouin’s solution	Sigma	Cat# HT10132
cyclohexanone	Sigma	Cat# 29140
n-Propanol	Sigma	Cat# 34871
O-(2,3,4,5,6-pentafluorobenzyl)hydroxylamine (PFBHA)	Sigma	Cat# 76735
formaldehyde 16%	ThermoFisher Pierce	Cat# 28906
Puregene cell lysis solution	QIAGEN	Cat# 1126462
Puregene protein precipitation solution	QIAGEN	Cat# 1126468
proteinase K	Fisher BioReagents	Cat# BP1700-100
NaCNBD_3_	Alfa Aesar	Cat# 087839.06
ultra-pure water	Romil	Cat# H949M
shrimp alkaline phosphatase	New England Biolabs	Cat# M0371
snake venom phosphodiesterase I	Sigma	Cat# P3243
DNase I	Roche	Cat# 04716728001
RNase A solution		Cat# 1014858
propidium iodide	Invitrogen	Cat# P3566

**Chemicals, peptides, and recombinant proteins**		

NC13	Chemometec	Cat# 910-3013
DNA Extract solution	Applied Biosystems	Cat# 4403319
QIAamp DNA Micro kit	QIAGEN	Cat# 56304
Quick-DNA MiniPrep	Zymo	Cat# D3025
ddPCR Supermix for Probes (no dUTP)	BioRad	Cat# 186-3023
21XMouse chromosome paints	MetaSystemsProbes	Cat# D-0425-120-DI
10x Chromium reagent kit v3	10x Genomics	https://www.10xgenomics.com/
QIAamp DNA Micro Kit	QIAGEN	Cat# 56304

**Deposited data**		

ScRNAseq data	This paper	GEO: GSE316966
Murine genomic sequencing	This paper	ENA: PRJEB107797

**Experimental models: Cell lines**		

32D		RRID:CVCL_0118

**Experimental models: Organisms/strains**		

*Adh5*^*tm1Stam*^	Gift from Limin Liu	MGI ID: 3033711
*Fancd2*^*tm1Hou*^	Gift from Markus Grompe	MGI ID: 2673422
*Adh5*^*tm1c(EUCOMM)Wtsi*^	EUCOMM	MGI ID: 6390883
*Adh5*^*tm1d(EUCOMM)Wtsi*^	EUCOMM	MGI ID: 6390884
*Commd10*^*Tg(Vav1-icre)A2Kio*^	JAX	MGI ID: 2449949
*Adh5*^*tm1a(switch)Kjpl*^	This study	MGI ID: 8262561 or 8262564

**Oligonucleotides**		

See Table S5	This study	N/A

**Software and algorithms**		

Zen	Zeiss	
FIJI	Schindelin et al.^73^	RRID:SCR_002285
MassHunter GCMS Acquisition Version B.07.05.2479	Agilent	N/A
MassHunter Quantitative Analysis Version B.07.01 SP1/Build 7.1.524.1	Agilent	N/A
Cytovision M-FISH v7.7	Leica	N/A
Cell Ranger pipeline (v6.0.1)		RRID:SCR_017344
Stardist	Schmidt et al.^74^	N/A
Interphase telomere analysis pipeline	Pixel Biology Ltd	N/A
CLIJ	Haase et al.^75^ and Vorkel and Haase^76^	N/A
MorphoLibJ	Legland et al.^77^	RRID:SCR_027135
BioVoxxel 3D Box	https://zenodo.org/badge/latestdoi/434949702	N/A
Emptydrops	Lun et al.^78^	N/A
Scanpy2	Wolf et al.^79^	N/A
Scrublet3	Wolock et al.^80^	N/A
TrimGalore		RRID:SCR_011847
Fastp	Chen^81^	RRID:SCR_016962
gatk cleansam v4.0.9.0		RRID:SCR_001876
bwa mem v0.7.12	Li and Durbin^82^	RRID:SCR_010910
samtools sort v1.15.1	Danecek et al.^83^	RRID:SCR_002105
gatk markduplicates v4.0.9.0		RRID:SCR_001876
samtools index v1.15.1	Danecek et al.^83^	RRID:SCR_002105
gatk Mutect2 v.4.2.0.0		RRID:SCR_001876
ANNOVAR		RRID:SCR_012821
bedtools intersect v.2.24.0	Quinlan and Hall^84^	RRID:SCR_006646
strelka v.2.9.2	Kim et al.^85^	N/A
gatk PileUp v.4.2.0.0		RRID:SCR_001876
bcftools v.1.16	Danecek et al.^83^	RRID:SCR_005227
HMMcopy v1.38.0 for R v.4.2.0		RRID:SCR_026464
circlize v.0.4.16 for R v.4.2.1	Gu et al.^86^	RRID:SCR_002141
delly v.0.8.7	Rausch et al.^87^	RRID:SCR_004603
manta v.1.6.0	Chen et al.^88^	RRID:SCR_022997
Keggrest v1.36.3	Tenenbaum and Maintainer^89^	RRID:SCR_026949
COSMIC GRCh38 v.95		RRID:SCR_002260
IntOGen v.022020	Martínez-Jiménez et al.^90^	N/A
PANTHER v19.0	Mi et al.^91^	RRID:SCR_004869
deconstructSigs v.1.8.0 for R v.4.2.1		RRID:SCR_026286
Turing package v0.29.1	Fjelde et al.^92^ and Ge et al.^92^	N/A
Julia v1.9.3		RRID:SCR_021666
GraphPad Prism v.10		RRID:SCR_002798
FlowJo v.10		RRID:SCR_008520

**Other**		

70 μm filter	Greiner	Cat# 54207
K_3_EDTA MiniCollect tubes	Greiner	Cat# 45031
Microvette 500 Z-gel tubes	Sarstedt	Cat# 20.1344
glass crimp top vials	Agilent	Cat# 5182-0543
magnetic crimp caps	Agilent	Cat# 5188-5386
7 mm stainless steel metal ball	QIAGEN	Cat# 69990
tissue lyser	QIAGEN Retsch	N/A
2000 MWCO Vivacon® 500	Sartorius	Cat# VN01H92
nanoEase M/Z Symmetry C18 Trap Column, 100Å, 5 μm, 180 μm × 20 mm	Waters	N/A
EASY-Spray HPLC analytical column 2 μm particle size, 75 μm × 250 mm	ThermoFisher Scientific	N/A
Cy3-T2AG3 PNA probe	Eurogentec	Cat# PN-TC050-005
X-VIVO medium	Lonza	Cat# 04-448Q
KaryoMAX™ Colcemid™	ThermoFisher Scientific	Cat# 15212012

### Experimental model and study participant details

#### Patient consent and materials

Informed consent and authorization for research and sample storage were given by FA patients or their relatives, in accordance with the Declaration of Helsinki and French law. IRB approval from INSERM was given under the number 12–078 to the overall human FA research project. Patient samples were obtained and cryopreserved from blood, bone marrow aspirate or skin biopsy at FA diagnosis evaluation of the patients, Paris, under the authorization CPP 1208 RIPH3.

#### Mice

All animals were maintained in specific pathogen-free conditions. Both sexes of mice were used. All animal experiments undertaken in this study were done so with the approval of the University of Oxford Animal Welfare Ethical Review Body and under project license authority, PP2564045, granted by the UK Home Office under the Animal (Scientific Procedures) Act 1986 License PFC07716E (1st March 2021–25th September 2023) or PP2564045 (20th September 2023–19th September 2028).

For all compound mutants, two inbred parental strains were generated and crossed to generate F1 hybrid experimental animals. *Adh5*^*tm1Stam*^ (MGI ID: 3033711) were a gift from Limin Liu and *Fancd2*^*tm1Hou*^ (MGI ID: 2673422) mice were a gift from Markus Grompe. *Adh5*^*tm1Stam*^ and *Fancd2*^*tm1Hou*^ mice were bred and maintained as previously described in Pontel et al.^25^ The *Adh5*^*tm1c(EUCOMM)Wtsi*^ allele (MGI ID: 6390883, abbreviated *Adh5*^*c*^), a conditional allele which is fully functional, was derived from breeding Flp recombinase into the *Adh5*^*tm1c(EUCOMM)Wtsi*^ EUCOMM mouse to generate offspring with the *tm1c* allele.^93^ This was bred with the *Fancd2*^*tm1Hou*^ strain to generate *Adh5*^*c/c*^ *Fancd2*^*+/−*^ mice and these were backcrossed to a 129S6/SvEvTac (hereafter 129) background for 10 generations. The *Adh5*^*tm1d(EUCOMM)Wtsi*^ allele (MGI ID: 6390884, abbreviated *Adh5*^*d*^ or *Adh5*^*d*^) was generated by crossing a Stella-Cre mouse with the *Adh5*^*tm1c(EUCOMM)Wtsi*^ mouse to generate the *tm1d* allele. *Adh5*^*d/d*^ mice are full *Adh5* knockouts. The *Fancd2*^*tm1Hou*^ and *Commd10*^*Tg(Vav1-icre)A2Kio*^ (MGI ID 2449949, abbreviated *Vav1-iCre*, purchased from Jackson Labs)^42^ alleles were bred into the *Adh5*^*tm1d(EUCOMM)Wtsi*^ strain and these mice were backcrossed onto a C57BL/6J (hereafter C57) background for 10 generations. To generate F1 hybrid experimental animals, males from the 129 *Adh5*^*c/c*^ *Fancd2*^*+/−*^ strain were crossed with female C57 *Adh5*^*d/d*^ *Fancd2*^*+/−*^ *Vav1-iCre*^*+ve*^ (due to leakiness of *Vav1-iCre* in sperm^94^) to generate *Adh5*^*c/d*^ *Fancd2*^*+/−*^ *Vav1-iCre*^*+ve*^ (referred to as *Adh5*^*c/-*^ *Fancd2*^*+/−*^ *Vav1-iCre*^*+ve*^ mice for ease of communicating which allele is the knockout). To generate *Adh5*^*tm1a(switch)Kjpl*^ (abbreviated *Adh5*^*s*^^*w*^; ^*MGI ID 8262561*^) mice, mouse embryonic stem cells carrying the *Adh5*^*tm1c(EUCOMM)Wtsi*^ allele were targeted with a vector containing exon 3 of *Adh5* in reverse orientation, flanked by modified Lox66 and Lox71 sites.^39^ A neomycin resistance cassette was positioned at the 3’ end. This construct was flanked by FRT sites and homology arms for targeting into the *Adh5* locus. ES cell clones were selected with neomycin, validated by PCR, then injected into blastocysts. Resultant chimeric mice were crossed with FLP-expressing mice, thereby inducing germline removal of the neomycin cassette in resultant pups, which were *Adh5*^*sw/+*^. These were intercrossed and resultant *Adh5*^*sw/sw*^ mice maintained through interbreeding. C57 *Adh5*^*sw/sw*^ mice were crossed with 129 *Fancd2*^*+/-*^ mice and backcrossed for 10 generations to generate 129 *Adh5*^*sw/+*^ *Fancd2*^*+/-*^ mice. *Adh5*^*sw/+*^*Fancd2*^*+/-*^ mice were intercrossed to generate *Adh5*^*sw/sw*^ *Fancd2*^*+/-*^ mice. Male 129 *Adh5*^*sw/sw*^ *Fancd2*^*+/-*^ mice were crossed with female C57 *Adh5*^*c/d*^ *Fancd2*^*+/−*^ *Vav1-iCre*^*+ve*^ mice to generate *e*xperimental *Adh5*^*sw/-*^*Fancd2*^*-/-*^ *Vav1-iCre* and controls. ADH5 and FANCD2 do not exhibit haploinsufficiency in mice, so *Adh5*^*sw/-*^ *Fancd2*^*+/-*^ or *Adh5*^*sw/-*^*Fancd2*^*+/-*^ *Vav1-iCre* mice were experimentally grouped with *Adh5*^*sw/-*^ *Fancd2*^*+/+*^ or *Adh5*^*sw/-*^ *Fancd2*^*+/+*^ *Vav1-iCre* mice and referred to as *Adh5*^*sw/-*^ or *Adh5*^*sw/-*^*Vav1-iCre* henceforth. Similarly, *Adh5*^*sw/+*^*Fancd2*^*+/+*^*, Adh5*^*sw/+*^*Fancd2*^*+/+*^ *Vav1-iCre, Adh5*^*sw/+*^*Fancd2*^*+/-*^, and *Adh5*^*sw/+*^*Fancd2*^*+/-*^ *Vav1-iCre* mice were simplified as wild-type (WT) for experiments. *Adh5*^*sw/+*^*Fancd2*^*-/-*^ or *Adh5*^*sw/+*^*Fancd2*^*-/-*^ *Vav1-iCre* mice were simplified as *Fancd2*^*-/-*^.

For transplantation experiments, CD45.1 B6SJL (B6.SJL-Ptprca Pepcb/BoyJ) and C57 (CD45.2) mice were purchased from Biomedical Services, University of Oxford. CD45.1/.2 mice were obtained by breeding B6SJL mice with C57 mice.

#### Genotyping

Mice ear biopsies were genotyped in-house and later, by Transnetyx, as detailed in Table S5.

### Method details

#### ddPCR confirmation of *Adh5* restoration in *Adh5*^*sw*^

DNA was extracted from mouse peripheral blood cells using DNA Extract solution (4403319 Applied Biosystems) according to manufacturer’s instructions. DNA from Fluorescence-Activated Cell Sorted granulocytes (see below; **Flow Cytometry and Cell Sorting**) was extracted using the QIAamp DNA micro kit (56304 QIAGEN). For controls, DNA was extracted from wildtype 32D cells, an *Adh5*^*tm1d/tm1d*^ mouse ear notch, or *Adh5*^*sw/sw*^ mouse spleen cells using Quick-DNA MiniPrep (D3025 Zymo). Prior to ddPCR, the DNA was digested using HindIII-HF (R3104S NEB) according to the manufacturer’s instructions. ddPCR was performed using ddPCR Supermix for Probes (no dUTP) (186-3023 BioRad) according to the manufacturer’s instructions. Three separate assays were constructed to recognise the Cre-recombined version of the *Adh5*^*sw*^ allele, using a FAM-conjugated internal probe, forward primers which sit outside the recombined region, and reverse primers within the recombined region, such that amplification can only occur if the sequence is the recombined-orientation. Primers and a HEX-conjugated probe targeting mouse *Trfc* were used as a reference assay for copy-number variation (CNV) calculation.

#### Histology

Spleens, femurs and sternii were fixed in 10% neutral-buffered formalin for a minimum of 24 hours. Femurs and sternii were decalcified in EDTA. Tissues were embedded in paraffin. After sectioning at 3.5 μm, tissues were stained using hematoxylin and eosin. Images were collected using a color camera (Infinity 3s, Lumenara) mounted on an Olympus BX60 microscope. Scale bars were added in FIJI.^95^

#### Blood Counts

Total blood was collected in K_3_EDTA MiniCollect tubes (45031 Greiner) and analyzed using a scil VetABC Plus+ blood counter (Horiba).

#### Cytospins

Bone marrow cells were isolated from femurs, tibiae, humeri, iliac crests, and occasionally spines using FACS buffer (PBS supplemented with 1% w.v. BSA [A4503 Sigma Aldrich]) and strained through 70 μm mesh (54207 Greiner). Spleen cell suspensions in FACS buffer were prepared by gently pressing and straining spleen through a 70 μm mesh (54207 Greiner). Cell suspensions from either source were spun unto slides using a Cytospin 4 (Thermo Scientific) and then stained using modified Wright stain (06689653 [Hematek Stain Pak] Hematek) on an automated staining platform (Hematek Bayer Health Care).

#### Embryo whole-mount microscopy and analysis

Whole-mount immunofluorescence on embryos was performed as previously described.^38^^,^^96^ Specifically, developmentally equivalent embryos (as assessed by somite count and vessel architecture) were stained using goat anti-CD31 (1:400; AF3628 R&D Systems followed by Alexa 488 donkey anti-goat IgG A1105 Invitrogen) and rat anti-C-KIT (1:200; 14-1171-85 eBioscience, followed by Donkey anti-Rat IgG (H+L) Alexa Fluor™ 568; A78946 Invitrogen). Images were acquired on a Zeiss LSM 900 upright microscope using a Zeiss C-Achroplan 32x/0.85 W Corr objective. Imaging was performed at room temperature. Images were processed using Zeiss Zen. 3D reconstructions are maximum intensity projections.

#### Flow Cytometry and Cell Sorting

##### HSC and progenitor staining and quantification

Bone marrow cells were isolated from femurs, tibiae, humeri, iliac crests, and occasionally spines using FACS buffer (PBS supplemented with 1% w.v. BSA [A4503 Sigma Aldrich]) and strained through 70 μm filter (54207 Greiner). Red cells were lysed by resuspending the cells in 10 mL hemolytic buffer (155 mM NH4Cl, 10 mM KHCO3, 0.1 mM Na2EDTA, pH 7.2) for 10 min at 4 °C. After centrifugation the cell pellet was resuspended in FACS buffer and nucleated cells were stained with NC13 (910–3013 Chemometec) and counted on Nucleocounter NC-3000 (Chemometec). 10 × 10^6^ bone marrow cells were resuspended in 200 μL of FACS buffer containing the following antibody solution: FITC-conjugated lineage cocktail with antibodies against CD3e 145-2C11 (eBioscience 35-0031-U500), CD4 H129.19 (BD 130308), CD8a 53-6.7 (BD 553031), Mac-1 (CD11b) M1/70 (BD 553310), CD11c (eBioscience 11-0114-85), B220 (CD45R) RA3-6B2 (BD 553088), FceR1a MAR-1 (eBioscience 11-5898-85), Gr-1 (Ly-6G) RB6-8C5 (BD 553127), Ter119 (BD 116206) and CD41 FITC (BD 553848), c-kit (CD117) APC eF780 (eBioscience 47-1171-82), Sca-1 PE-Cy7 clone D7 (eBioscience 25-5981-82), CD150 BV785 (BioLegend 115937), CD48 Biotin (BioLegend 103410), Strep BV421 (BioLegend 405225), Flt3 (CD135) PE A2F10 (eBioscience 12-1351-82), CD34 AF700 (eBioscience 56-0341-82), CD16_32 (FcgRII/III) APC (eBioscience 17-0161-82) and IL7Ra (CD127) BV605 (BioLegend 135025) with viability assessment using 7-AAD (BioLegend 420404). Analysis was performed on LSR Fortessa X20 (Becton Dickinson).

##### Mature lineage staining and quantification

Bone marrow cells (10 × 10^6^), prepared as above, were resuspended in 200 μL FACS buffer containing a mature lineage cocktail consisting of antibodies against: CD4 BV421 Clone H129.19 (BD 740024), CD8a BV786 Clone 53_6.7 (BD 563332),Gr1/Ly-6G FITC RB6-8C5 (eBioscience 553127), B220/CD45R APC Clone RA3-6B2 (BD 553092),CD3e BV605 Clone 145-2C11 (BD 563004), Ter119 PE-Cy7 Clone Ter119 (BD 557853), IgM APCef780 Clone II/41 (eBioscience 47-5790-82), CD71 PE Clone R17 217.1.4 (eBioscience 12-0711-82), Mac/CD11b BV711 M1/70 (BD 563168). Spleen cell suspensions in FACS buffer were prepared by gently pressing and straining whole spleen through a 70 μm mesh (54207 Greiner). Populations in the peripheral blood were quantified by red cell lysing 100 μL of whole blood with addition of 1 mL of hemolytic buffer incubated for 10 min at 4 °C and washed with 3 mL of FACS buffer. Following centrifugation, cells were resuspended in 100 μL FACS buffer containing the mature lineage cocktail. Ter-119 was used to exclude unlysed red cells and red cell debris.

#### Competitive Repopulation Assay

Briefly, CD45.1/.2 recipients were subjected to two doses of 4.5 Gy whole-body irradiation, 3 hours apart, before intravenous injection of a cell suspension containing 1e6 nucleated fetal liver CD45.2^+^ cells from donor and 200,000 sex-matched nucleated bone marrow cells from a CD45.1^+^ competitor. Chimerism in the peripheral blood was determined at 4 weekly intervals after red cell lysis using a cocktail containing antibodies against CD4 and CD8a (FITC-conjugated, as above), B220 PerCP-Cy5.5 (BioLegend 103236), CD11b PE (BD 553311), Gr-1 PE (BioLegend 108407), Ter119 PE-Cy7 (Invitrogen 25-5921-82), CD45.1 BV421 (BioLegend 110732) and CD45.2 APC (BioLegend 109814). Donor-derived chimerism was calculated as the fraction of CD45.2^+^ CD45.1^-^ cells among the sum of CD45.1^+^ CD45.2^-^ and CD45.2^+^ CD45.1^-^ cells in a population.

#### Western Blots

ADH5 antibody (rabbit polyclonal, clone 48^51^) was used at 1:1000 in 2% w/v non-fat milk powder (NFM; A0830,0500 PanReac AppliChem), 1× TBS, 0.05% Tween-20 at 4°C with gentle shaking, overnight. Vinculin antibody (mouse monoclonal, clone V284; 05-386 EMD Millipore) was used at 1:20,000 in the same conditions. Swine anti-rabbit immunoglobulins HRP (P0217 Dako) was used as secondary antibody at 1:1,000 for ADH5 or goat anti-mouse HRP (31432 Invitrogen) at 1:5000 for Vinculin, for 2 hours at room temperature.

#### Micronucleus Assay

Micronucleus assay was performed as previously described previously.^97^ Blood (20 μL) from mice 6-40 weeks of age (mean 15 weeks) was added to 110 μL solution of heparin in PBS (1000 U/ mL). 120 μL of the blood suspension were added to 1.2 mL methanol at −80°C and stored for at least 12 hours at −80°C. Fixed blood was washed and resuspended in bicarbonate buffer (0.9% (w/v) NaCl, 5.3 mM NaHCO3). Blood in bicarbonate buffer (a volume equivalent to 2 μL blood) was incubated with 1 μL anti-CD71 FITC, clone R17217.1.4 (11-0711-82 eBioscience), 7 μL RNase A (1014858 Sigma) in a total volume of 100 μL for 45 min, washed with 1 mL bicarbonate buffer, and resuspended in 500 μL of a 5 μg/mL solution of propidium iodide in bicarbonate buffer and analyzed immediately.

#### CFU-S Assay

CFU-S assays were performed as described previously by Garaycoechea et al.^27^^,^^97^ Briefly, total bone marrow was flushed from femora and tibiae. Nucleated cells were counted using a solution of 3% acetic acid and methylene blue and nucleated bone marrow cells (1x10^5^ for controls and 1x10^6^ for *Adh5*^*c/-*^ *Fancd2*^*-/-*^ *Vav1-iCre*) were intravenously injected into lethally irradiated recipients. After 12 days, spleens were isolated and fixed in Bouin’s solution (HT10132 Sigma), the total number of colonies was counted and expressed relative to the number of total bone marrow cells injected.

#### Serum Formaldehyde Quantification by GC-MS

This is as described in Dingler et al.^47^ and is summarized as follows. Whole blood from cardiac puncture (500 μL-700 μL) was collected into Microvette 500 Z-gel tubes containing clotting activator (20.1344 Sarstedt). After centrifugation at 10,000 × g for 5 min at room temperature, 100 μL of serum was transferred to glass crimp top vials (5182-0543 Agilent), followed by addition of internal standards: cyclohexanone (29140, Sigma) and n-Propanol (34871 Sigma) at a final concentration of 1 mg/ mL each respectively, and derivatization reagent O-(2,3,4,5,6-pentafluorobenzyl)hydroxylamine (PFBHA) (76735 Sigma) at a final concentration of 60 μg/mL. After sealing with magnetic crimp caps (5188-5386 Agilent), the tubes were incubated overnight in the dark at room temperature, and then stored at −80 °C until analysis by GC–MS. A serum-formaldehyde calibration standard was prepared in parallel with each batch of serum sample collection as follows. Dilutions of formaldehyde 16% (w/v) (28906 ThermoFisher Pierce) in PBS were added to cardiac drawn blood at final concentrations ranging from 0 μM–213 μM. Subsequent serum isolation and formaldehyde derivatization was identical to sample preparation as described above.

The mass spectrometer was operated in single ion monitoring mode for the ions m/z 181, 195 and 225 for formaldehyde-PFBHA oxime (retention time 11.47 min) and m/z 181, 195 and 293 for cyclohexanone-PFBHA oxime internal standard (retention time 16.73 min) with m/z 181 used for quantification for both compounds. A dwell time of 200 ms was used for each ion. The transfer line to the mass spectrometer was heated to 220 °C, the source temperature was maintained at 230 °C and the quadrupole at 150 °C. The GC–MS data were acquired using MassHunter GCMS Acquisition B.07.05.2479. For quantification, all analyte integrated peak areas were ratioed to internal standard areas using MassHunter Quantitative Analysis Version B.07.01 SP1/Build 7.1.524.1 for GCMS. The method was calibrated across the range of 0.1 to 5 mg l−1 of formaldehyde: each calibration point was run in triplicate and a demonstrated precision of ≤ 15%.

#### Extraction of DNA from tissue samples for LC-MS analysis

Tissue samples were snap-frozen and stored at -70 °C until analysis. 10-20 mg of tissue was lysed in a 2 mL reaction tube (72.695 Sarstedt) in 730 μL of Puregene cell lysis solution (1126462 QIAGEN), 4 μL of proteinase K (BP1700-100 Fisher BioReagents™, 20 mg/mL in H_2_O) with a 7 mm stainless steel ball (69990 QIAGEN). Samples were homogenized in a tissue lyser (QIAGEN Retsch) for 4 min at 30 Hz, then incubated at 37 °C for 30 min, 600 rpm (using an Eppendorf ThermoMixer). Then 4 μL of RNase A solution (1014858 QIAGEN) was added, vortexed and incubated at 37 °C for 1 hour at 600 rpm.

The supernatant was transferred to a new tube (1.7 mL, Axygen) and cooled on ice for 1 min. Then 265 μL Puregene protein precipitation solution was added (1126468 QIAGEN), vortexed briefly, and spun 21,300 × g, 3 min, 4 °C. The supernatant was transferred into a fresh tube containing 600 μL ice cold isopropanol, mixed by inversion 10× and left at RT for 5 min for the DNA to precipitate. DNA was pelleted at 21,300 × g for 2 min, 4 °C. The supernatant was discarded and the DNA pellet washed with 600 μL of 70 % ethanol, spun at 21,300 × g, 2 min, 4 °C. Again, the supernatant was discarded and the pellet left to air-dry for 5 min.

#### Reduction of purified DNA pellets for LC-MS analysis of adducts

The purified DNA pellet was dissolved by addition of 500 μL of 50 mM NaCNBD_3_ in 200 mM NaOAc (pH = 5.2, diluted from 3M stock, S7899 Sigma), and left for 48 hours at 37 °C at 900 rpm in an Eppendorf ThermoMixer.

DNA was precipitated by addition of 900 μL ice cold isopropanol and spun at 21,300 × g, 5 min, 4 °C. The was supernatant discarded. This step was repeated with 70 % ethanol and the pellet left to air dry. The DNA was dissolved overnight at RT in 80 μL of ultra-pure water (H949M Romil) and then quantified by Nanodrop One (Thermo Scientific).

#### DNA digestion for LC-MS

DNA was digested in a total volume of 100 μL in reactions containing 10 μg DNA, 2U shrimp alkaline phosphatase (M0371 New England Biolabs), 0.004 U snake venom phosphodiesterase I (P3243 Sigma) and 10 U DNase I (04716728001 Roche) in 1 × DNase I digestion buffer.

Also added to all digests were the internal standards ^15^N-*N*^*2*^-Me-dG and ^15^N-dA. For standard curve generation, a non-reduced sample of DNA isolated from WT liver (10 μg) was used and standards for 2′-deoxyadenosine (dA) and *N*^2^-MeD-dG were added at various concentrations. The range of the standard curves was as follows: 21 to 690 nmol for dA and 0.09 to 5 fmol for *N*^2^-MeD-dG. The curves contained 6 points plus a zero control (H_2_O in place of standards). The response ratio (peak area of analyte to labelled internal standard) was plotted vs the amount of analyte standard injected onto the column.

After overnight digestion (>16 h), samples were centrifuged at 21,300 × g, 2 min, and the top 50 μL carefully transferred to a MS vial (186000385c Waters) and analyzed.

#### LC-MS^2^ determination of N_2_-MeG in DNA digests

Samples were analyzed on TSQ Altis Triple Quadrupole Mass Spectrometer in selected reaction monitoring mode (SRM) interfaced to an UltiMate 3000 uHPLC. The uHPLC was fitted with an Acclaim PepMap C18 column (2 μm particle size, 300 μm × 15 cm, Thermo Fisher Scientific) connected to an EASY-Spray™ source at 35 °C via an EASY-Spray™ cap flow emitter, 15 μm.

2 μL of sample (100 ng of digested DNA on column) was injected per run using a 5 μL sample loop. Solvents used were from Romil and of Ultra LC standard. Solvent A: H2O (0.1% acetic acid), solvent B MeCN (0.1% acetic acid). The standard gradient used was 0-2.5 min – 1 % B, 26.5 min – 12.5 % B. This was followed by 2 wash pulses (1-90 % B) and equilibration to 1 % B (45 min total run time).

Mass spectrometry conditions were as follows: source voltage of 2000V in positive ionization mode; ion transfer tube temperature 275 °C, CID gas pressure 1.5 mTorr, scan widths for Q1 and Q3 at 0.7 *m*/*z*, a chromatographic filter was used with a peak width of 6 sec. Collision energy voltage and RF voltage were optimized with authentic standards using the vendor-provided Tune software for each fragment in the SRM, however the dA parameters were reduced to 10 % of the optimal value due to their very large peak size.

Data was analyzed using the FreeStyle 1.8 software and Genesis peak detection algorithm, or Tracefinder 4.1.

#### M-FISH

M-FISH was performed on methanol–acetic acid-fixed murine bone marrow material using commercially available, combinatorially labeled, whole chromosome 21XMouse chromosome paints (D-0425-120-DI MetaSystems Probes) according to the manufacturer's protocol. Images were collected using Leica HC PL FLUOTAR 100x/1.32 objective mounted on a Leica DM6000B microscope equipped with appropriate filters and analyzed using Cytovision M-FISH v7.7 software (Leica).

#### Telomere FISH

PNA FISH was carried out as previously described.^98^ Specifically, telomere specific signals were detected using a Cy3-T2AG3 PNA probe (PN-TC050-005 Eurogentec) hybridised to methanol:acetic acid (v:v 3:1) fixed cells from whole bone marrow aspirate. Bone marrow aspirate was cultured *ex vivo* for 1h in X-VIVO medium (Lonza) containing 10 uL/ mL of KaryoMAX™ Colcemid™ (15212012 ThermoFisher Scientific). Images were collected using a widefield fluorescence DeltaVision Elite system (Applied Precision) equipped with an Olympus UPLSAPO 100 × /1.40 oil immersion objective, a CoolSnap HQ2 CCD camera (Photometrics), DAPI (excitation 390/18; emission 435/40) and TRITC (excitation 542/27; emission 593/45) filters. 12-bit image stacks were acquired with a z-step of 200 nm giving a voxel size of 64.5 nm x 64.5 nm x 200 nm. Metaphases were often limited or absent.

#### Sorting specific cell populations for WGS

##### Human samples

DNA was prepared from CD34^+^ cells, enriched by bead purification from BM material and fibroblast cells, expanded from skin biopsy, from the same patient was taken as a germline reference.^59^ Sequencing libraries were prepared by Novogene and sequenced as a service on Illumina NovaSeq X Plus by Novogene.

##### Murine samples

Spleen cells (for eventual collection of B cells) and bone marrow cells (for eventual collection of granulocytes) were stained with the mature lineage cocktail as described in section **Flow Cytometry and Cell Sorting**. Cells (2 x 10^5^-1 x 10^6^) were sorted into PBS on FACSAria Fusion (BD). Brain cortex material from the same animal was taken as a germline reference. DNA was prepared using Qiagen QIAamp DNA Micro Kit. Sequencing libraries were prepared by Novogene and sequenced on platform Illumina NovaSeq6000 by Novogene.

#### WGS data processing

For murine data, raw sequencing reads from sorted granulocyte, B cells and matched cortex control samples were 5’ and 3’ trimmed using either Trim Galore, a wrapper for cutadapt, or fastp. Reads were mapped to the murine mm10 reference genome using bwa mem v0.7.12. For human data, raw sequencing reads from bone marrow samples and matched fibroblasts control samples were 5’ and 3’ trimmed using fastp. Reads were mapped to human hg19 reference genome with bwa mem v.0.7.12. In both cases, bam files were cleaned with gatk cleansam v4.0.9.0 and coordinate-sorted using samtools sort v1.15.1. Duplicate reads were marked with gatk markduplicates v4.0.9.0 and bam files were indexed using samtools index v1.15.1.

#### Variant calling

For murine data, somatic SNVs (sSNVs) and indels in granulocyte and B cell samples were called using gatk Mutect2 v.4.2.0.0 in tumor-normal mode, with matched cortex samples serving as germline controls. Variants were filtered by internal gatk Mutect2 filters, setting the minimum median mapping quality to 30 and applying a read-orientation filter. All variants were annotated with ANNOVAR according to the murine reference genome version mm10. Variants located in repeat regions and simple repeat regions (defined by regions downloaded from https://genome.ucsc.edu/cgi-bin/hgTables, selecting "mm10" > Variation & Repeats > "RepeatMasker", "SimpleRepeats") were filtered using bedtools intersect v.2.24.0. VAFs of sSNVs and indels were calculated as the number of variant reads divided by the sum of variant reads and reference reads. For human data, sSNVs and indels in bone marrow samples were called with gatk Mutect2 v.4.2.0.0 in tumor-normal mode using matched fibroblast samples as germline control. Variants were filtered by internal gatk Mutect2 filters, setting min-median-mapping-quality to 30 and applying a read-orientation filter. All variants were annotated with ANNOVAR according to the human reference genome version hg19. Variants in repeat regions and simple repeat regions (regions downloaded from https://genome.ucsc.edu/cgi-bin/hgTables selecting “hg19” > Variation & Repeats > “RepeatMasker”, “SimpleRepeats”) were filtered using bedtools intersect v.2.24.0. VAFs of sSNVs were calculated as the number of variant reads divided by the sum of variant reads and reference reads. Detected sSNVs were confirmed by a second somatic SNV caller (strelka v.2.9.2).

#### Post-call filtering of sSNVs and indels in murine data

Accounting for the hybrid strain background of C57BL/6 and 129S of the mice, we developed an additional filter to remove technical artefacts in sequencing data. All SNVs that occurred at an unexpected frequency in multiple mice were removed from our analysis. Specifically, for each granulocyte sample, the VAFs of all detected SNVs were counted in the raw sequencing data of all samples from other mice within our dataset using gatk PileUp v.4.2.0.0. If the detected VAF of a SNV in the sequencing data of other mice exceeded an acceptable baseline VAF, it was removed from the analysis. The acceptable baseline VAF was calculated per base substitution in trinucleotide context in the following way: the frequency of each possible base substitution class in trinucleotide context was counted in >50 unrelated murine WGS samples at 100 random sites per class. Confidence intervals of observed frequency values per random site were calculated per base substitution class and served as reference baseline. In this way, we account in our filtering that a certain fraction of variant reads recurred randomly across mice. Any detected SNV in our data with a VAF higher than the upper limit of the reference baseline's 95% confidence interval in other mice was excluded from further analysis. SNVs at sites of copy number alterations or loss of heterozygosity mutations were removed from VAF based analysis, confining the interpretation of VAF histograms to heterozygous diploid regions. Further, we excluded SNVs with less than three variant reads or which are listed in a murine SNP database (doi:10.1186/s13059-016-1024-y). For SNVs covering gonosomes in male individuals the VAF was corrected to a pseudo-diploid VAF. To increase the precision of indel calling, for each mouse the presence of all detected indels was evaluated in all other mice of the same dataset. For this, the raw pre-filtering output of Mutect2 was evaluated with bcftools v.1.16. If the same indel was called in another mouse, it was removed from further analysis. All remaining indels were confirmed by a second somatic variant caller (strelka v.2.9.2^85^ (https://doi.org/10.1038/s41592-018-0051-x). Indels that are listed in a murine SNP database^99^ were excluded from further analysis.

#### Copy number alterations

Read count distributions were created using HMM copy utils and the R package HMMcopy v1.38.0 for R v.4.2.0. Read counts were corrected for GC content and mappability according to the murine mm10 or human hg19 reference genomes. Number of bins along genome was randomly subsampled and plotted in Circos plots using the R package circlize v.0.4.16 for R v.4.2.1.

#### Loss of heterozygosity mutations

For the detection of loss of heterozygosity mutations, heterozygous germline SNVs and indels were detected by running gatk Mutect2 v.4.2.0.0 in tumor-only mode for granulocytes samples and their matched germline control samples. Default internal Mutect2 filters were applied to all detected variants. Heterozygous variants detected in germline controls samples were filtered for minimum sequencing coverage of 10, mapping quality of variant and reference allele = 60, median base quality of variant and reference allele = 37 and 0.4 < VAF < 0.6. VAF of heterozygous germline variants detected in germline control samples was evaluated in granulocyte samples and plotted in Circos plots using the R package circlize v.0.4.16 for R v.4.2.1.

#### Structural variants

Somatic structural variants (SVs) in mouse granulocyte samples were called using delly v.0.8.7 with matched germline control in tumor-normal mode. We excluded default regions of complex structure and high sequence similarity of mm10 reference genome provided by delly. We applied default delly filters. Pre-filtered somatic sites were further profiled and filtered across a larger panel of control samples (five cortex samples from within the dataset) to efficiently filter false positives and germline SVs. Remaining SVs were recalled with manta v.1.6.0. If the same type of structural variant (deletion/ insertion/ inversion/ translocation) detected by delly was detected by manta within a 1000bp radius around the breakpoints identified by delly, it was included in further analysis. All remaining structural variants of VAF >.2 were plotted in circos plots using the R package circlize v.0.4.16 for R v.4.2.1. For the human data, we analysed SVs for patient EGF325 with clonal hematopoiesis, calling them using delly v.0.8.7 in tumor-normal mode. We excluded default regions of complex structure and high sequence similarity of hg19 reference genome provided by delly. We applied default delly filters. Pre-filtered somatic sites were further profiled and filtered across a larger panel of control samples (29 hair samples from an unrelated human dataset) to efficiently filter false positives and germline SVs. Remaining SVs were recalled with manta v.1.6.0. If the same type of structural variant (deletion/ insertion/ inversion/ translocation) detected by delly was detected by manta within a 1000bp radius around the breakpoints identified by delly, it was included in further analysis.

#### Search for potential clonal hematopoiesis driver genes

Murine sSNVs and indels identified by primary calling of indels and snvs (Mutect2 output, before applying stricter filters) were annotated using the mm10 gene database provided by ANNOVAR. Variants annotated as “exonic” and “not synonymous”, as well as genes affecting CNAs and SVs, were further examined. The affected (murine) genes were checked for relevance in an FA-related pathway. To this end, the genes presence was evaluated in a database (Keggrest v1.36.3) for genes relevant in: Fanconi anemia pathway, aldehyde pathway, nucleotide excision repair pathway, homologous recombination pathway or non-homologous end joining pathway. Further, affected murine genes were translated in human orthologues via an orthologue catalogue (v. 2022, downloaded from https://www.informatics.jax.org/homology.shtml). Human genes were checked for presence in two catalogues of known human driver genes (COSMIC GRCh38 v.95 and IntOGen v.022020). Additionally, genes were checked for presence on Haem p53Score^41^ list consisting of 16 *Trp53* target genes that exhibit p53-dependent pattern of expression in LKS cells. Affected genes were also checked against the WES, WGS and NGS data in generated from a large French fanconi anemia patient cohort. In addition, murine regions affected by CNAs were also checked for synteny to the human 1q duplicated region reported within the same publication.^59^ Affected genes were additionally checked against sequencing data generated from fanconi anemia revertants and patients by the same group (unpublished results). We also cross-referenced all genes affected by exonic non-synonymous variants, indels, SVs and CNAs against genes with identified variants found in aged normal mice^21^ and genes exhibiting clonality in a 115 year-old individual without apparent drivers.^13^ Genes affected by sSNVs, indels, SV, and CNAs were checked for pathway clustering by using PANTHER pathways (PANTHER v19.0 https://pantherdb.org ). Genes affected by indels and SVs were checked for CH drivers in the same way as genes affected by sSNVs.

Similarly, in human WGS data, exonic sSNVs and indels detected by Mutect2 before intersecting with Strelka^85^ were checked for covering genes present in a database (Keggrest v1.36.3) for genes relevant in: Fanconi anemia pathway, aldehyde pathway, nucleotide excision repair pathway, homologous recombination pathway or non-homologous end joining pathway. Further, the genes were checked for presence in two catalogues of known human cancer driver genes (COSMIC GRCh38 v.95 and IntOGen v.022020). Further, for CNAs and structural variants, murine genes affected by copy number alterations or structural variants were detected using the mm10 gene database provided by ANNOVAR^100^ and translated to human orthologues (catalogue of orthologues v. 2022). Human genes were checked for presence in two catalogues of known human driver genes (COSMIC GRCh38 v.95 and IntOGen v.022020). For 5.8 y FANCA patient, genes affected by structural variants were detected using the hg19 gene database provided by ANNOVAR and checked for presence in two catalogues of known human driver genes (COSMIC GRCh38 v.95 and IntOGen v.022020).

#### Mutation Signature Plots

Signature plots of sSNVs were created with R package deconstructSigs v.1.8.0 for R v.4.2.1. For the murine data, SNV data was merged by genotype.

#### MATHEMATICAL MODELING

##### Inference from VAF distribution with SCIFER

Population-dynamic parameters were inferred from granulocyte variant allele frequencies (VAFs) using the previously described method SCIFER (**s**elected **c**lone **i**n**fer**ence).^20^ Briefly, stem cells undergo an expansion phase followed by homeostasis while constantly acquiring sSNVs. For parameter inference we use the homeostatic phase modeled by SCIFER as a practical approximation of to slow stem cell attrition indicated by the experiments (for an explicit stem cell attrition model, see below). The resulting site frequency spectrum *S*_n_(*t*) – the number of variants found in *n* cells at time *t*, is given by

$$S_{n}\left(t\right)=\int_{0}^{t}\mu \;\lambda \left(\tau \right)\;N\left(\tau \right)\;p_{n}\left(\tau ,t\right)\;d\tau$$

where *λ*(*τ*) is the cell division rate (which assumes a constant value *λ*_exp_ during expansion and a potentially different constant value *λ*_ss_ during homeostasis), *μ* is the number of mutations a cell acquires per cell division, *N*(*τ*) is the population size at intermediate time points *τ* (which first grows exponentially and remains constant during homeostasis at size *N*_ss_), and *p*_n_(*τ*,*t*) is the probability that a clone (defined by a newly acquired somatic variant) that emerged at an intermediate time point *τ* has grown to size *n* by time *t*. This probability is obtained from the theory of birth-death processes.^20^ The VAFs relate to the clone size *n* via $\text{VAF}=n/\left(2\;N_{\text{SS}}\right)\text{.}$

Finally, the experimental error of sequencing is accounted for by assuming sequencing-depth-dependent variation of the observed VAFs around the true VAFs according to a beta distribution.

Assuming the emergence of a selected clone at time *t*_S_, SCIFER allows for the inference of five parameters: *μ*, *λ*_ss_, *N*_ss_, *t*_S_, and the selective advantage *r*. Parameter inference was conducted using Approximate Bayesian Computation, where the prior distributions were taken as uniform. The units and upper and lower bounds for mice were taken as:

**Table:** 

Parameter	Unit	Bounds
$\log_{10}\left(\mu \right)$	mutations per cell division	(-2, 3)
$\log_{10}\left(\lambda_{\text{ss}}\right)$	1/week	(-2, 1.5)
$\log_{10}\left(N_{\text{ss}}\right)$	–	(1, 6)
$t_{S}$	week	(0, t)
r	–	(0, 1)

The mouse data comprise in total 14 samples: 3 aged WT mice, 1 aged *Adh5*^*c/-*^ *Vav-iCre* mouse, 1 aged *Fancd2*^*-/-*^ *Vav-iCre* mouse, 2 young *Adh5*^*c/-*^ *Fancd2*^*-/-*^ *Vav-iCre* mice, 5 aged *Adh5*^*c/-*^ *Fancd2*^*-/-*^ *Vav-iCre* mice (including one mouse that escaped *Adh5* deletion). Since only the 5 aged *Adh5*^*c/-*^ *Fancd2*^*-/-*^ *Vav-iCre* mice exhibited obvious clonal mutations, we used SCIFER with selected clones for these samples, while the data of all other mice were described without selected clones.

For humans, the following prior specifications were used:

**Table:** 

Parameter	Unit	Bounds
$\log_{10}\left(\mu \right)$	mutations per cell division	(-2, 3)
$\log_{10}\left(\lambda_{\text{ss}}\right)$	1/year	(0, 3)
$\log_{10}\left(N_{\text{ss}}\right)$	–	(1, 8)
$t_{S}$	year	(0, t)
r	–	(0, 1)

Since only the 5.8y *FANCA* individual exhibited obvious clonal mutations, we used SCIFER with selected clones for this subject, while the data of the 3 other individuals were described without selected clones.

##### Stem cell attrition model and fitting of the Kaplan-Meier curve

In order to relate the time points of death of the *Adh5*^*c/-*^ *Fancd2*^*-/-*^ *Vav-iCre* mice (Kaplan-Meier curve) to stem cell attrition, we considered a subcritical birth-death process and assumed that the time of death corresponds to the time point when stem cells became extinct. To this end, we assumed that for each mouse the number of stem cells at birth is *N*_0_. Each stem cell may divide with rate *λ* and is subject to loss (death and/or differentiation) with rate *δ*, where subcritical implies *δ* > *λ*. Under this condition, the system will go extinct within finite time. The probability that such a system has survived up to time *t* is

(Equation 1) $$\begin{array}{c} p_{S}\left(t\right)=1-{\left[\frac{e^{\left(1-\xi \right)\delta t}-1}{e^{\left(1-\xi \right)\delta t}-\xi }\right]}^{N_{0}} \end{array}$$

where we defined the subcritical parameter *ξ* = *λ*/*δ*. Thus, the model comprises three parameters: *N*_0_, *δ*, and *ξ*. We used the measured Kaplan-Meier curve to estimate the model parameters via Bayesian inference. Specifically, we used NUTS sampling with an acceptance rate of 0.45 to obtain posterior distributions with 1000 samples via the Turing package (v0.29.1) in Julia (v1.9.3). We assumed the uncertainties in the data to be normally distributed with standard deviation σ, to be estimated alongside the other model parameters. The model prior distributions were chosen uniform with the following specifications:

**Table:** 

Parameter	Unit	Bounds
$\log_{10}\left(N_{0}\right)$	–	(0, 3)
δ	1/week	(0.01, 10)
ξ	–	(0, 1)
σ	–	(0, 1)

In a second step, we used the inferred parameters to simulate VAF distributions resulting from the attrition model. To properly account for the large expected variability in clone sizes in the attrition process, we performed stochastic simulations. The simulations operate on single-cell resolution, where each cell is characterized by a vector of integer-valued mutation indices. If a cell divides, this mutation vector is copied to both daughter cells, where the mutation vector of each daughter is appended by a novel mutation index (infinite-sites hypothesis). This enables the degree of mutation sharing within the cell population to be tracked and to eventually determine the site frequency spectrum. The simulations were set up such that the cell population starts with a somatically unmutated cell at time *t*_0_ and, during development, expands to population size *N*_0_ with division rate *λ*_e_ (supercritical birth-death process without loss), which is reached at time *t*_B_. As the resulting site frequency spectrum is independent of the precise value of *λ*_e_ we set *λ*_e_ = 1. From *t*_B_ onward the population follows a subcritical birth-death process, with division rate *λ* and loss rate *δ* > *λ*. Sampling parameter values from the posterior distributions of the fit of the Kaplan-Meier curve for the simulations therefore assures that simulated mice follow the same survival statistics as *Adh5*^*c/-*^ *Fancd2*^*-/-*^ *Vav-iCre* mice. In total, we simulated 100 mice, where we computed the site frequency spectrum at 5, 25, 30 and 35 weeks after birth, given the simulated mouse was still alive (number of BFAs > 0).

### Quantification and statistical analysis

#### Telomere FISH analysis

In order to assess relative telomeric length we therefore developed an interphase-based analysis approach. A two-step analysis pipeline was used. The pipeline is as follows: nuclei were segmented using a marker-controlled watershed using the segmentation of a 2D Sobel projection by Stardist^74^ as seeds and intensity thresholded mask as a binary restriction mask. Telomeres were segmented using marker-controlled watershed using the maximas of the small hessian eigenvalue as seeds and the product of the small and medium hessian eigenvalue as binary restriction mask. The analysis was performed in FIJI, using primarily the CLIJ, MorphoLibJ and BioVoxxel 3D Box libraries.^73^^,^^75^^,^^76^^,^^77^^,^^95^^,^^101^

#### General Statistical Analysis

Sample number (n) indicates the number of independent biological samples in each experiment and are indicated in figure legends or methods. Statistical tests undertaken and significances achieved are given in figures, figure legends or methods. Unless otherwise stated in the figure legends, data are shown as the mean ± SEM. Analysis was performed using GraphPad Prism (v.10) and FlowJo (v.10).

#### scRNA-seq data analysis

Single-cell sequencing libraries were generated using 10x Chromium (10x Genomics, Pleasanton, CA) reagent kit v3 according to the manufacturer’s protocol and sequenced on an Illumina Novaseq 6000 platform. Raw reads were mapped to the mm10 genome and quantified using the Cell Ranger pipeline (v6.0.1) with default parameters. Cell-associated barcodes and background-associated barcodes were determined using the EmptyDrops method^78^ implemented in the Cell Ranger pipeline, and the background-associated barcodes were excluded. Subsequent data analysis was performed using Scanpy2.^79^ Multiplets were estimated using the Python package Scrublet3^80^ and subsequently removed. Cell libraries with less than 1,500 detected genes or with mitochondrial gene expression exceeding 10% of UMI counts were further removed. The gene expression matrix was log-normalized, and 2,000 highly variable genes were identified using the Scanpy function ‘pp.filter_genes_dispersion’. Cell cycle scores were computed for each cell using a previously published list of cell cycle-associated genes4 and the Scanpy function ‘tl.score_genes_cell_cycle’. The cell cycle scores, total UMI counts and mitochondrial gene percentages were then regressed out using the Scanpy function ‘pp.regress_out’. The expression values of the highly variable genes were scaled and used to compute 50 principal components. The principal components were subsequently used to identify 15 nearest neighbors and to compute clusters and the UMAP embedding using the Scanpy functions ‘tl. louvain’ and ‘tl.umap’, respectively. The clusters were annotated manually using known marker genes for each lineage. The p53 score was calculated using the Scanpy function ‘tl.score_genes’, as previously defined.^41^

## Supplemental information

Document S1. Figures S1–S6

Table S1. Parameter estimate details, related to Figure 5 Showing parameter estimates for BFA counts, division rates (1/week), time to fixation, mutation rate (1/week), and time of BFA clone in murine granulocytes and B cells.

Table S2. Post-filter sSNV lists, related to Figure 5 Curated post-filtered sSNVs from BM granulocyte sequencing data underlying the graphs, from aged *Adh5*^*c/*−^*Fancd2*^−*/*−^*Vav1-iCre* animals, shown in Figures 5F and S3C.

Table S3. Murine lists, related to Figure 5 Listing the sSNVs, found in each aged *Adh5*^*c/*−^*Fancd2*^−*/*−^*Vav1-iCre* animal, used to interrogate driver status. These are exonically mapping sSNVs, retrieved from pre-filtered data, that may functionally affect the gene. VAFs >5% were checked against COSMIC for cancer causation status and IntOGen for CH driver status.

Table S4. FA patient sSNV Lists, related to Figure 6 Listing the sSNV used to interrogate whether known drivers were present in Fanconi anemia patients, as shown in Figures 6 and S6.

Table S5. Genotyping, related to STAR Methods Primer details for standard and ddPCR.

Document S2. Article plus supplemental information
