# Supplementary material for: Metabolite-induced DNA damage drives stochastic stem cell loss and clonal hematopoiesis
Source: Cell Stem Cell. 2026 Apr 2;33(4):642–659.e11. doi: 10.1016/j.stem.2026.02.011 (PMC7619061; doi:10.1016/j.stem.2026.02.011)
Supplement: Document S1. Figures S1–S6 [file mmc1.pdf]

Cell Stem Cell, Volume *33*

Supplemental Information

Metabolite-induced DNA damage drives stochastic stem cell loss and clonal hematopoiesis

Ashley N. Kamimae-Lanning, Jill M. Brown, Matthias Günther, Franziska Esau, Holly Russell, Lise Larcher, Frédéric Langevin, Tomoya Isobe, Nicola K. Wilson, Felix A. Dingler, Rebecca L. Cordell, Meng Wang, Christopher L. Millington, Nina Claudino, Ewa Gogola, Matthew Nicholls, Verena Körber, Berthold Göttgens, Marella F. T.R. de Bruijn, Juan I. Garaycoechea, Jean Soulier, Thomas Höfer, and Ketan J. Patel

Figure S1: Murine models used lacking formaldehyde detoxification and DNA repair in the hematopoietic system, related to Fig. 1, 2, 3

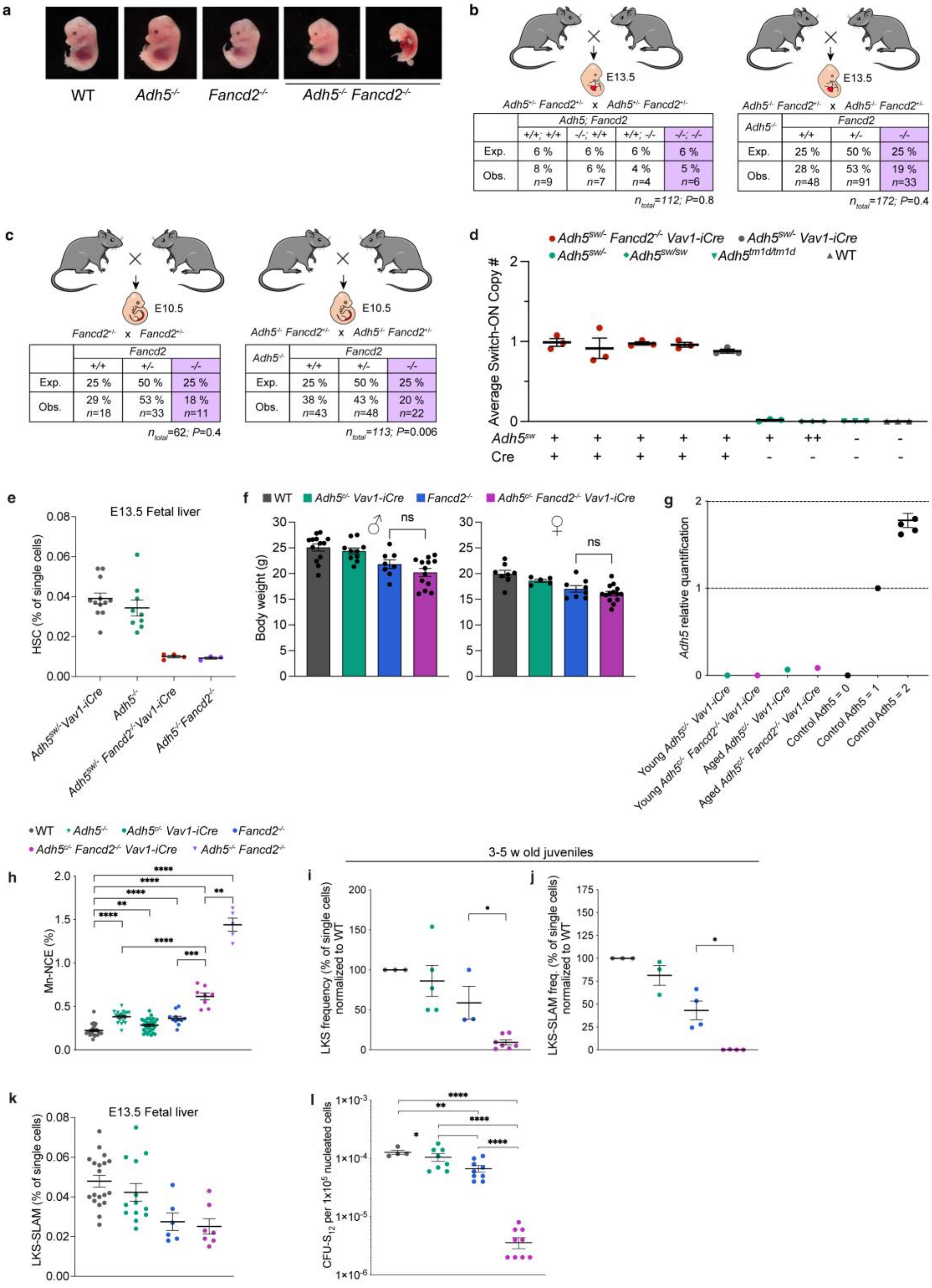

Figure S1: Murine models used lacking formaldehyde detoxification and DNA repair in the hematopoietic system, Related to Figures 1, 2 and 3.

a) Representative images of E13.5 *Adh5*$^{-/-}$ *Fancd2*$^{-/-}$ embryos and genotypic controls.
b) *Adh5*$^{-/-}$ *Fancd2*$^{-/-}$ E13.5 fetal Mendelian ratios table from two alternate crossing approaches. Intermediate genotypes not shown. n= total number of embryos and *P* value is by Fisher's exact test.
c) *Adh5*$^{-/-}$ *Fancd2*$^{-/-}$ E10.5 fetal Mendelian ratios table from two alternate crossing approaches. n= total number of embryos genotyped, and *P* value is by Fisher's exact test.
d) Digital droplet PCR results showing almost complete restoration (0.92-0.99) of the *Adh5*^{sw} allele in the blood of mice by *Vav1-iCre.* When *Vav1-iCre* is absent, the allele remains unswitched.
e) Flow cytometry quantification of fetal liver HSCs in E13.5 embryos for *Adh5*$^{sw/-}$ *Vav1-iCre, Adh5*$^{sw/-}$ *Fancd2*$^{-/-}$ *Vav1-iCre* and controls (left graph; 1 dot per embryo, n = 12, 9, 5, 3, from left to right, mean ± SEM).
f) Weights of male (left graph) and female (right graph) mice of the given genotypes at 6 weeks of age (mean ± SEM).
g) Adh5 qPCR in blood of young and aged *Adh5*$^{c/-}$ *Vav1-iCre* and *Adh5*$^{c/-}$ *Fancd2*$^{-/-}$ *Vav1-iCre* mice.
h) Percentage of micronucleated erythrocytes, as quantitated by flow cytometry analysis, in peripheral blood from *Adh5*$^{c/-}$ *Fancd2*$^{-/-}$ *Vav1-iCre* mice and controls. Triangles indicate global *Adh5* deletion. Each dot/triangle represents data from one mouse. n_{blood} analyzed = 23, 19, 29, 11, 8, 5 from left to right, mean ± SEM.
i) Flow cytometry quantification of bone marrow LKS cells (immunophenotypic HSPCs) in juvenile mice (left graph; 1 dot per mouse except for WT which is averaged in each experiment, n_{WT}=6, $n_{Adh5c/-\ Vav1\text{-}iCre}$=5, $n_{Fancd2-/-}$=3, $n_{Adh5c/-\ Fancd2-/-\ Vav1\text{-}iCre}$=7, mean ± SEM; equivalent data for adult mice shown in Fig 3g).
j) Flow cytometry quantification of bone marrow LKS-SLAM cells (immunophenotypic HSCs) in juvenile mice (1 dot per mouse except for WT which is averaged in each experiment, n_{WT}=6, $n_{Adh5c/-\ Vav1\text{-}iCre}$=3, $n_{Fancd2-/-}$=4, $n_{Adh5c/-\ Fancd2-/-\ Vav1\text{-}iCre}$=4, normalized to WT average within each experiment, mean ± SEM; equivalent data for adult mice shown in Fig. 3h).
k) Flow cytometry quantification of *Adh5*$^{c/-}$ *Vav1-iCre, Adh5*$^{c/-}$ *Fancd2*$^{-/-}$ *Vav1-iCre* and controls (right graph; 1 dot per embryo, n = 19, 13, 6, 7, mean ± SEM; equivalent data for adult and juvenile mice shown in Fig. 3h and Supp. Fig. 1h, respectively).
l) Repeat experiment for 12-day CFU-S assay, complementing that shown in Fig. 3i. n = 4, 8, 9, 10, from left to right, mean ± SEM.

Unless otherwise stated, *P*-values were determined by two-tailed Mann-Whitney U test, * *P*<0.05, ** *P*<0.01, *** *P*<0.001 **** *P*<0.0001.

Figure S2: Splenic hematopoiesis and scRNA-seq, related to Figure 4

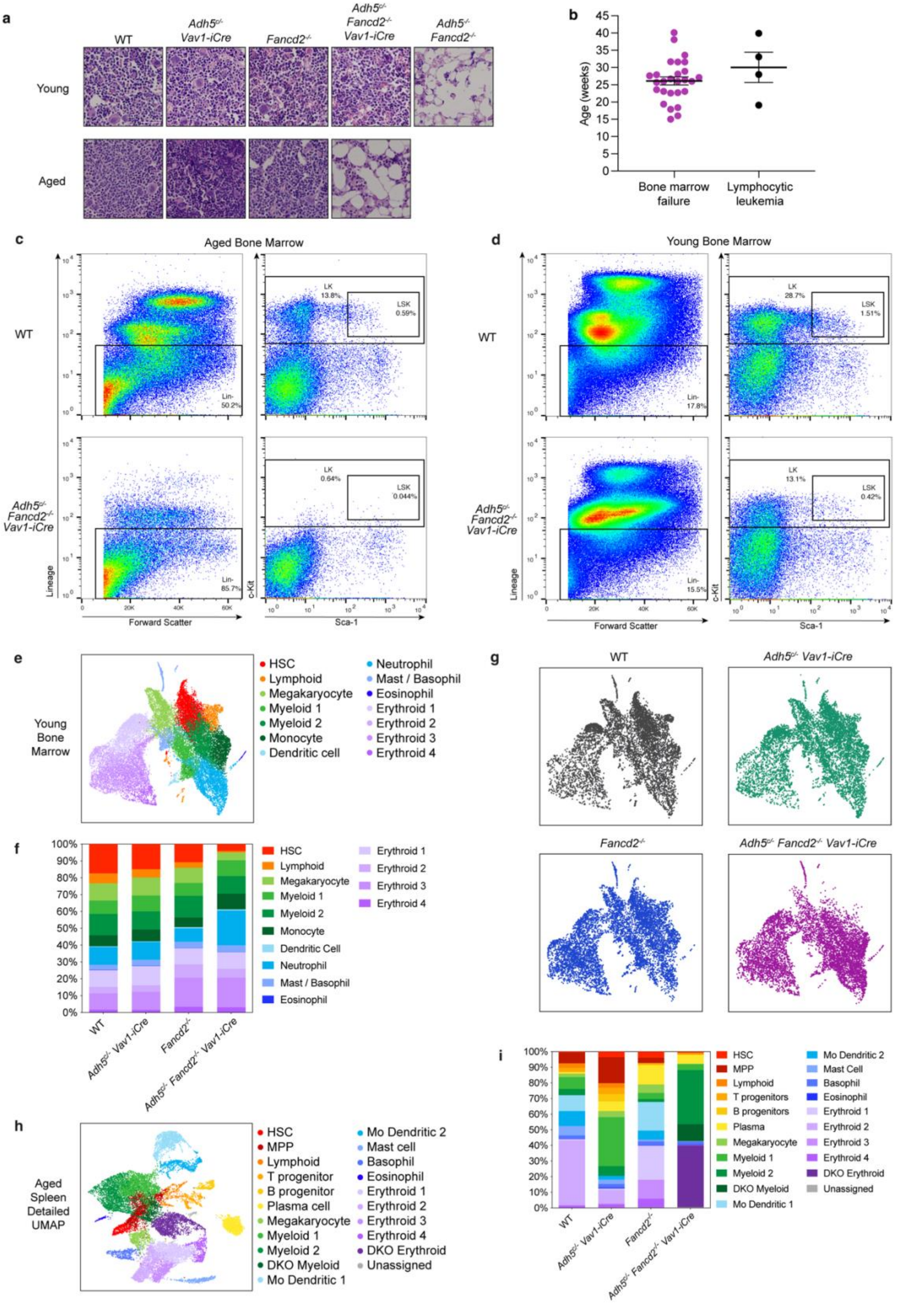

Figure S2: Splenic hematopoiesis and scRNA-seq, related to Figure 4

a) H&E-stained bone marrow sections from young and aged mice. Young *Adh5*$^{-/-}$ *Fancd2*$^{-/-}$ (global double mutant) and aged *Adh5*$^{c/-}$ *Fancd2*$^{-/-}$ *Vav1-iCre* bone marrow sections both show considerable aplasia.
b) Graph showing age at death, by cause, of *Adh5*$^{c/-}$ *Fancd2*$^{-/-}$ *Vav1-iCre* animals as shown in Kaplan-Meier analysis (Fig. 4a). Most deaths (27/31 animals; 87%) in *Adh5*$^{c/-}$ *Fancd2*$^{-/-}$ *Vav1-iCre* animals were a result of bone marrow failure (purple dots).
c) Representative flow cytometry plots of Lin^{-} c-Kit^{+} (LK) gating from aged bone marrow from WT and *Adh5*$^{c/-}$ *Fancd2*$^{-/-}$ *Vav1-iCre* animals.
d) Representative flow cytometry plots of Lin^{-} c-Kit^{+} (LK) gating from young bone marrow from WT and *Adh5*$^{c/-}$ *Fancd2*$^{-/-}$ *Vav1-iCre* animals.
e) UMAP plot of single-cell 10X RNA-seq on LK gated cell transcriptomes, from bone marrow of young mice. All genotypes are superimposed, with colors representing detailed lineage clusters.
f) Proportions of cells from each detailed lineage cluster from single-cell transcriptomic analysis of LK sorted cells from young bone marrow.
g) UMAP visualization of LK transcriptomes from young bone marrow cells, colored according to each genotype, shown separately to highlight differences in proportions of clusters.
h) A detailed clustering version of the UMAP from aged spleen LK cells shown in Fig. 4e.
i) Proportions of cells from each detailed lineage cluster from single-cell transcriptomic analysis of LK cells from aged spleens.

Figure S3: Details relating to SCIFER and variant analyses, related to Figure 5

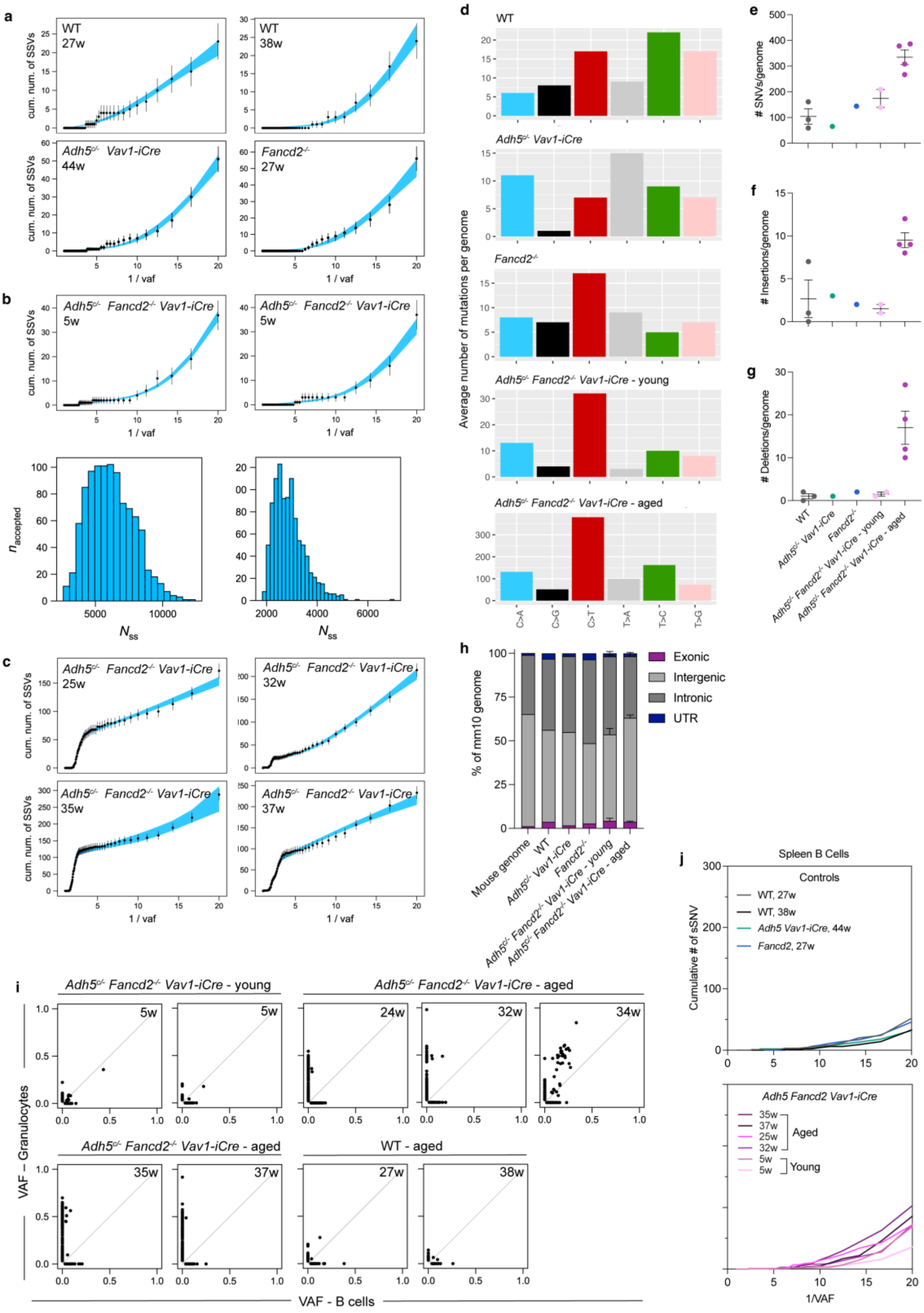

Figure S3: Details relating to SCIFER and variant analyses; related to Figure 5

a) Fitting of the SCIFER model to the cumulative somatic variant frequencies from individual mice of aged control mice (blue shading indicates 95% posterior probabilities of model fit).
b) Upper panels show model fit to 5-week-old *Adh5*$^{c/-}$ *Fancd2*$^{-/-}$ *Vav1-iCre* mice. Lower panels show posterior distributions for the number of BFAs as inferred by the SCIFER steady-state model for the same mice.
c) Model fit to aged *Adh5*$^{c/-}$ *Fancd2*$^{-/-}$ *Vav1-iCre* mice.
d) Counts of sSNVs (passing filters) in whole genome-sequenced granulocytes from aged control (n=3), young (n=2) and aged *Adh5*$^{c/-}$ *Fancd2*$^{-/-}$ *Vav1-iCre* mice *(n=4).*
e) Number of single nucleotide variants per animal in the shown genotypes (one spot per animal; mean ± SEM).
f) Number of insertions per granulocyte genome in the shown genotypes (one spot per animal; mean ± SEM).
g) Number of deletions per granulocyte genome in the shown genotypes (one spot per animal; mean ± SEM).
h) Genomic distribution of granulocytic somatic variants is the same across genotypes. Genomic proportions in mm10 mouse genome shown as reference (grouped by genotype. Mean ± SEM is shown).
i) Analysis of variants shared between or private to granulocytes and B cells.
j) Variant distribution in B220+ splenocytes from mice in Fig. 5d-f, indicating polyclonal production of these long-lived cells.

Figure S4: Parameter estimation for simulations of the attrition model, related to Figure 5

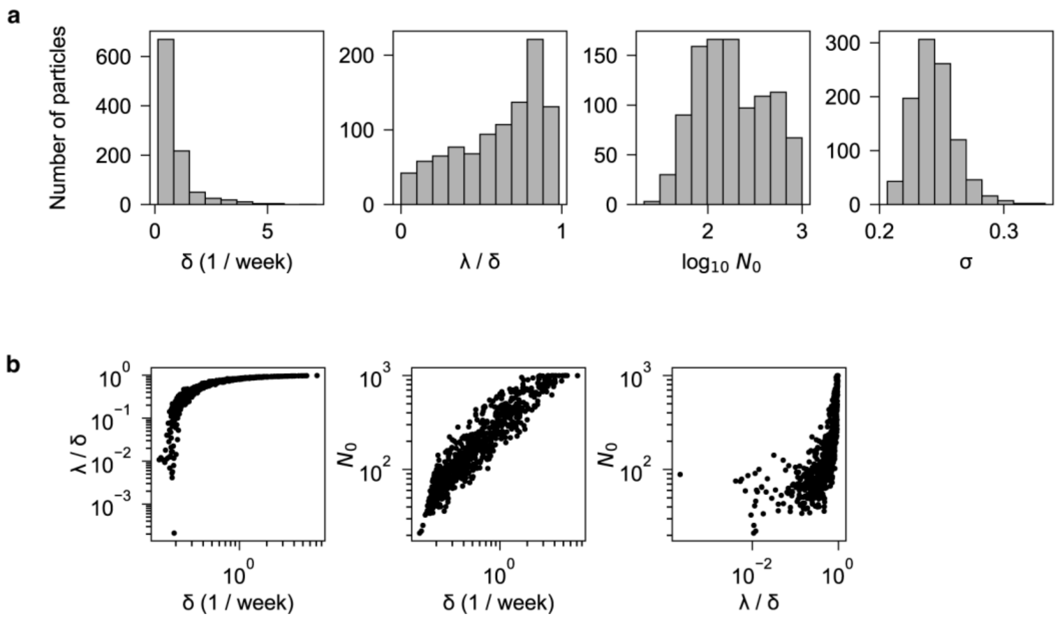

Figure S4: Parameter estimation for simulations of the attrition model, related to Figure 5

a) Posterior distributions of the model parameters obtained from fitting the attrition model to the experimental Kaplan-Meier data of the *Adh5*$^{c/-}$ *Fancd2*$^{-/-}$ *Vav-iCre* mice (Fig. 4a, Fig. 5g). The ages of death (from bone marrow failure) of *Adh5*$^{c/-}$ *Fancd2*$^{-/-}$ *Vav1-iCre* mice can fit an attrition-based model. λ = division rate, δ = loss rate, N_0 = initial cell number, σ = measurement uncertainty.

b) Correlations of the posterior distributions of the three mechanistically relevant model parameters.

Figure S5: Detection of translocation by M-FISH, loss of heterozygosity at *iCre* locus in one *Adh5^c/-^ Fancd2^-/-^ Vav1-iCre* mouse permits survival of intact Adh5 gene structure and telomere FISH, Related to Figure 5

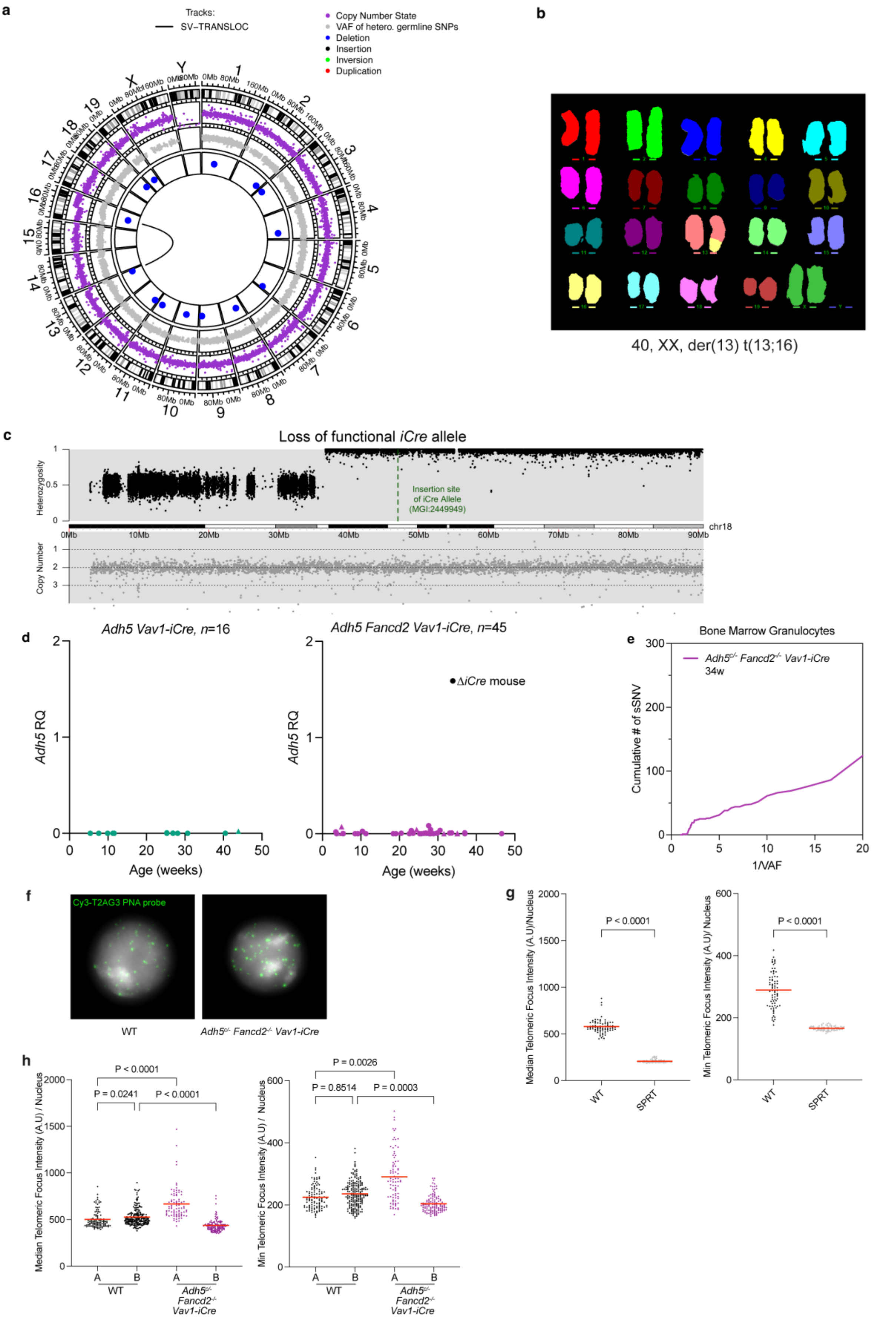

Figure S5: Detection of translocation by M-FISH, loss of heterozygosity at *iCre* locus in one *Adh5*$^{c/-}$ *Fancd2*$^{-/-}$ *Vav1-iCre* mouse permits survival of intact *Adh5* gene structure and telomere FISH, related to Figure 5

a) Circos plot of an aged *Adh5*$^{c/-}$ *Fancd2*$^{-/-}$ *Vav1-iCre* mouse that had a translocation of genetic material from chromosome 16 to 13.
b) M-FISH and resultant karyotype obtained from the same mouse in a).
c) Upper panel shows loss of heterozygosity on mchr18 region encompassing the *Vav1-iCre* locus in 34w *Adh5*$^{c/-}$ *Fancd2*$^{-/-}$ *Vav1-iCre* mouse. The bottom panel shows that mchr18 copy number remains the same across the entire length of the chromosome, confirming a neutral loss of heterozygosity status in this mouse. Plots are generated from sequence reads from BM granulocytic DNA. This 34w *Adh5*$^{c/-}$ *Fancd2*$^{-/-}$ *Vav1-iCre* mouse has therefore lost the *Vav1-iCre* locus and is referred to as Δ*iCre.*
d) A*dh5* qPCR on peripheral blood genomic DNA from control *Adh5*$^{c/-}$ *Vav1-iCre* and *Adh5*$^{c/-}$ *Fancd2*$^{-/-}$ *Vav1-iCre* mice, with Δ*iCre* mouse highlighted in area where copy number >1. RQ = relative quantification. Triangles represent mice used in WGS.
e) SCIFER analysis of bone marrow granulocytes from Δ*iCre* mouse, demonstrating clonal hematopoiesis due to selection of *Adh5*-proficient clone.
f) Representative images of telomere FISH probes in nuclei of bone marrow cells.
g) Comparison of telomeric foci intensities in interphase cells from BM preparations from age-equivalent F1 WT (long telomeres) and *M. spretus* (SPRT; short telomeres) (32.7wk and 25.9wk respectively) using a custom analysis pipeline on PNA TeloFISH preparations. Short telomeres (found in *M. spretus* mice) are robustly revealed by both median (left panel) and minimum telomeric intensity value per nucleus (right panel). WT n=76, SPRT n=112. Significance Kruskal-Wallis test. Means shown.
h) Using the telomere length assessment parameters described in b, two *Adh5*$^{c/-}$ *Fancd2*$^{-/-}$ *Vav1-iCre* mice with BMF (*Adh5*$^{c/-}$ *Fancd2*$^{-/-}$ *Vav1-iCre*-A and *Adh5*$^{c/-}$ *Fancd2*$^{-/-}$ *Vav1-iCre*-B; 30.3wk and 46.3wk respectively) were compared to their WT littermate controls (WT-A and WT-B; 30.3wk and 46.3wk respectively) and assessed for median (left panel) and minimum telomeric intensity value per nucleus (right panel). Comparing to the appropriate WT littermate control, median and minimum telomeric intensity values were seen to increase (in *Adh5*$^{c/-}$ *Fancd2*$^{-/-}$ *Vav1-iCre*-A) and decrease (in *Adh5*$^{c/-}$ *Fancd2*$^{-/-}$ *Vav1-iCre*-B). Replicative telomere shortening cannot therefore universally explain the BMF seen. WT-A n=186, WT-B n=201, *Adh5*$^{c/-}$ *Fancd2*$^{-/-}$ *Vav1-iCre*-A n=85, *Adh5*$^{c/-}$ *Fancd2*$^{-/-}$ *Vav1-iCre*-B n=126. Significance Kruskal-Wallis test. Means shown.

Figure S6: Fanconi anemia patient details, Related to Figure 6

a

Abbreviated *FANC* Mutations	Full mutations
FANCA CompHet	NM_000135.2(FANCA):c.3923T>A:p.Leu1308* & NM_000135.2(FANCA):c.367C>T:p.Glu123*
FANCA CompHet	NM_000135.2(FANCA):c.3391A>G:p.Thr131Ala & NM_000135.2(FANCA):c.3916T>G:p.Phe1306Val
FANCG CompHet	NM_004629.2(FANCG):c.1182_1192delinsC:p.Glu395Trpfs*5 & NM_004629.2(FANCG):c.1158del:p.Ser387Profs*16
FANCA Hom	homozygous del of exon 15 (NM_000135)

b

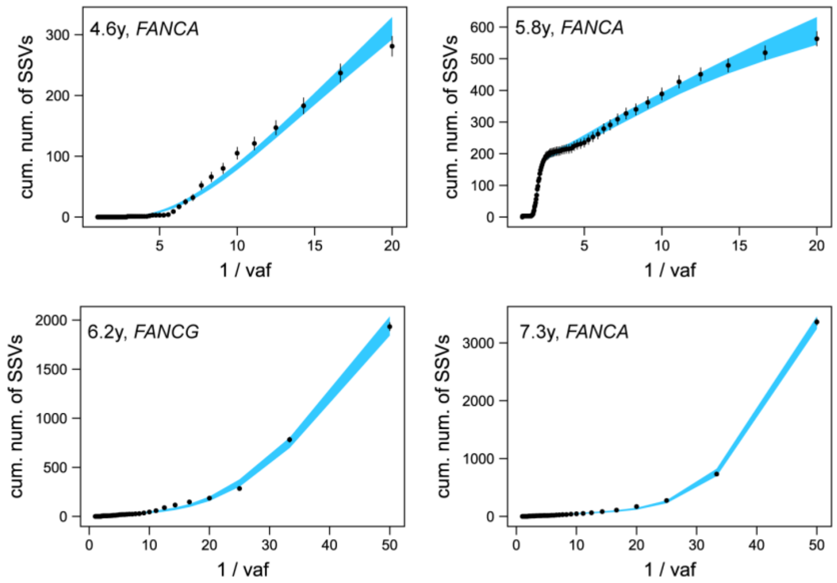

Figure S6: Fanconi anemia patient details, related to Figure 6

a) Complementation group gene mutation details for the Fanconi anemia patients included in this study.
b) Fitting of the SCIFER selection-based model to the cumulative somatic variant frequencies from deep whole genome sequenced CD34+ bone marrow from the four patients described (blue shading indicates 95% posterior probabilities of model fit).
